# New Discoveries Supporting the Exceptional Species Diversity of Opostegidae in Central America and the Caribbean, Alerting on Misidentified Barcodes #

**DOI:** 10.3390/insects16111170

**Published:** 2025-11-17

**Authors:** Jonas R. Stonis, Andrius Remeikis, Svetlana Orlovskytė

**Affiliations:** State Scientific Research Institute Nature Research Centre, Akademijos g. 2, LT-08412 Vilnius, Lithuania; remeikis.andrew@gmail.com (A.R.); s.orlovskyte@gmail.com (S.O.)

**Keywords:** molecular barcodes, Neotropics, Neotropical fauna, new species, *Pseudopostega*, species distribution, taxonomy

## Abstract

The aim of this study was to supplement current knowledge on the species diversity of Opostegidae in Central America and the Caribbean and to compare this diversity with that of other regions. We examined historical material and conducted fieldwork in Honduras during 2023–2025, a true *tabula rasa* in terms of Opostegidae diversity. Collected specimens were dissected, with genitalia photographed and analyzed. Molecular divergence was assessed using Neighbor-Joining and Maximum Likelihood methods, as well as Bayesian inference; creation of a mitotype network (TCS algorithm) and species delimitation (bPTP method) were also performed. The study of historical material revealed that *Pseudopostega saltatrix* (Walsingham) is not conspecific with taxa previously published under the same name, resulting in the description of one new *Pseudopostega* species. Fieldwork in Honduras yielded 11 additional *Pseudopostega* species—all new national records, six of which are new to science. The paper introduces 33 new molecular sequences, bringing the total to 114 mtDNA COI-5′ sequences currently deposited in the National Genomics Data Center (China). With these discoveries, the number of Opostegidae in Central America and the Caribbean rises to 63 species, representing 30.9% of the global fauna. The Neotropical realm (103 spp.) exhibits markedly higher Opostegidae diversity than other biogeographical regions, underscoring its importance as a center of diversification. Our analysis also revealed an alarmingly high proportion of doubtful molecular barcodes—nearly one-third (27%) appear erroneous due to species misidentification in Neotropical Opostegidae.

## Simple Summary

Species inventory is an important, engaging, and rewarding process that deepens our understanding of biodiversity. Previous studies have already highlighted the remarkable species diversity of white pygmy moths (Opostegidae) in the Neotropics. We hypothesize that, despite earlier research efforts, the taxonomic diversity in Central America exceeds current estimates. To test this, we examined historical material and carried out targeted fieldwork in Honduras, a true *tabula rasa* in terms of Opostegidae research. Its lack of prior studies, combined with its high ecological heterogeneity, made Honduras an ideal focal point. During our investigation, we discovered and described six new species, raising the number of Opostegidae known from Central America and the Caribbean to 63. This total represents nearly one-third of the global fauna and exceeds the entire tropical Oriental (Indomalayan) fauna, underscoring particularly strong diversification in the Neotropics. Our study also demonstrated that species richness remains unevenly documented among Neotropical countries, reflecting differences in sampling effort. Although the family itself can be recognized easily, species-level identification is far more difficult. Our analysis further indicated an alarming situation of misidentified DNA sequences, which create downstream problems in DNA barcoding databases.

## 1. Introduction

This paper focuses on Opostegidae (white eye-cap moths or white pygmies), a morphologically distinctive and phylogenetically basal family of Lepidoptera [1]. Together with their sister group, the pygmy moths (Nepticulidae) [2], they comprise the superfamily Nepticuloidea—one of the earliest-diverging monotrysian lineages in the order Lepidoptera [1,3,4,5,6,7].

Among the vast diversity of Lepidoptera, members of the family Opostegidae stand out as some of the most morphologically distinctive—and unmistakable—micro-moths. The adults of white pygmies usually ranging from just 4 to 4.7 mm in wingspan (with a minimum recorded wingspan of 3.7 mm) [8]. Most species are almost entirely white, exuding an elegant simplicity, with only sparse markings—typically limited to subtle strigulae, fine transverse lines of dark scales located at the forewing apex. Only a few notable exceptions exist worldwide, in which adults deviate from this pale palette and display fuscous or modestly patterned wings, predominantly in shades of pale brown or orange [9,10,11,12].

Like their closest relatives in Nepticulidae, opostegids bear a frontal tuft of piliform scales on the head; however, in contrast, the opostegid tuft is small and typically white, only occasionally tinged with ochre or brown. Another striking feature of these moths is their dorsoventrally flattened body, a rare trait among Lepidoptera. Equally remarkable is the greatly expanded antennal scape, which forms a conspicuous “eye cap” that not only exceeds the eye diameter but often entirely covers the eye when the moth is at rest—a characteristic that is only reduced or absent in the genus *Neopostega* Davis [13]. Perhaps most uniquely, the collar of Opostegidae sets them apart from all other Lepidoptera. This large, shield-like structure is composed of smoothly arranged lamellar scales and extends over the back of the head, often partially overlapping the thorax—an elegant morphological diagnostic feature of the group. As a result, a resting opostegid moth—whether on tree bark or illuminated on a light-trap screen at night—with its wings closed and scapes covering the eyes, resembles a tiny, distinctly oval-shaped, flattened, glossy white insect—something unmistakably unique among Lepidoptera, and indeed, unlike any other insect.

Regarding male genitalia, one of the most characteristic traits is the presence of a pedunculate cucullar lobe on the valva. This lobe bears a prominent pectinifer, consisting of a single row of blunt spines, and is attached to the valva by a slender, elongate pedicellum [10]. In the largest genus of Opostegidae, *Pseudopostega* Kozlov, the male genitalia completely lack a sclerotized phallus. In another highly speciose genus, *Opostegoides* Kozlov, the phallus is sclerotized only in part—often just a small portion—and is predominantly membranous. In females, the genitalia are distinctive. Unlike their sister family, Nepticulidae, which possesses both anterior and posterior apophyses, opostegid females are characterized by only a single pair, the posterior apophyses.

Comprehensive morphological treatments of Opostegidae have been presented in major revisions [2,9,13], and most recently by Davis and Stonis [10], forming the foundation for modern understanding of the family’s morphology and systematics.

While the family is readily distinguishable, species-level identification remains challenging due to the moths’ tiny size, subtle external variation, and minimal morphological differences between closely related species, necessitating genitalia dissection and expert analysis—issues we address later in the Discussion. The growing recognition of these diagnostic difficulties within Opostegidae has become increasingly relevant in recent years due to the expanding use of DNA barcoding and the consequent rise in misidentified sequences in public databases such as BOLD and GenBank. Because numerous, often very similar and presumably young species prevail in this family, the study of genitalia remains essential for accurate identification. Reliable diagnostics require careful attention to minute details, combined with substantial experience and the use of high-quality optics.

The biology of white pygmies remains largely unknown. To date, larval feeding habits have been studied for only a very small number of Opostegidae species, and our understanding of their life history remains fragmentary. Available data indicate that larvae of a few species are cambium miners [13,14], feeding within the living tissues of the inner bark—specifically the cambial layer—while a few others have been found as stem miners [2,15,16,17]. Biological information on the family was summarized by Davis [13], Davis and Stonis [10], and more recently briefly addressed by van Nieukerken et al. [18]. Interestingly, although immature stages are rarely observed—largely due to their highly concealed feeding habits—adults are frequently attracted to light traps in the Neotropics [19], suggesting that their true species richness and ecological diversity remain vastly underestimated.

The global diversity of Opostegidae remains enigmatic, particularly due to the family’s underrepresentation in many faunal surveys—most notably across tropical regions. Based on current data, including the new taxa presented in this study, the family is estimated to comprise 204 species worldwide—not including six Oriental species formally documented by Puplesis and Robinson [9] but still unnamed.

Although the family has received more sustained attention in boreal regions such as Europe and North America, they contribute significantly to fewer species than the tropics. Previous studies have already highlighted the rich species diversity of the Oriental (Indomalayan) region [9,20] and, especially, of the Neotropical realm. Within the latter, Central America—particularly Costa Rica—has been repeatedly identified as harboring the highest known species richness of Opostegidae worldwide [10,19,21]. The claim that Central America—particularly Costa Rica—is exceptionally rich in Opostegidae diversity raises a question: can we confidently say that the full taxonomic diversity of Opostegidae in this region has already been revealed? This is especially relevant given that the previous attention to this region was already remarkable.

Thus, in our investigation of white pygmy moth diversity in Central America and the Caribbean, we selected Honduras—the second largest country in Central America—as a focal point for a targeted study. It is worth noting that prior to our research, no studies on Opostegidae had ever been conducted in Honduras. As such, the country represented a true *tabula rasa* in terms of white pygmy moth diversity. This absence of prior research, combined with Honduras’ ecological heterogeneity and its relative proximity to the well-studied fauna of Costa Rica, made it an ideal setting for our investigation.

***The Working Hypothesis.*** We hypothesize that, despite previous research efforts, the taxonomic diversity of Opostegidae in Central America remains underestimated, and our focused investigation in Honduras—a single tropical country within one of the most extensively studied regions globally—can help quantify how much previously unrecognized diversity still remains.

***The Goal of the Study.*** The aim of this study was to supplement and consolidate data on the taxonomic diversity of Opostegidae in Central America and the adjacent Caribbean region. To achieve this, the following objectives were pursued: (1) examination of historical Opostegidae material housed in the collection of the Natural History Museum, London, including the first documentation of the type material of the little-known and taxonomically controversial Caribbean species *Pseudopostega saltatrix* (Walsingham), along with the description of a new species from the Caribbean; (2) based on fieldwork conducted in Honduras, identification of the species occurring in the country and description of taxa new to science; (3) summarizing the results and providing an updated checklist of Opostegidae species from the region with an expanded Neotropical coverage.

## 2. Materials and Methods

***Materials.*** The material examined in this study was collected by the first author in 2023–2025 in Honduras through two collaborative, long-term programs between the European Union and the Republic of Honduras (see Acknowledgments). All specimens reported in this paper will be deposited in the entomological collections of the Museum für Naturkunde (MfN), Berlin, Germany, following the publication of this study.

The type series of all previously described species included in our study were examined earlier by us in the respective museum collections, as detailed in the monograph on the American Opostegidae by D. R. Davis and J. R. Stonis [10]. Additionally, historical material from the Caribbean housed in the collections of the Natural History Museum, London (United Kingdom), was examined. This included the type series of *Pseudopostega saltatrix* from the U.S. Virgin Islands—comprising the male holotype and one male paratype—as well as two unidentified *Pseudopostega* specimens collected in Jamaica in 1905.

**Figure 1:**
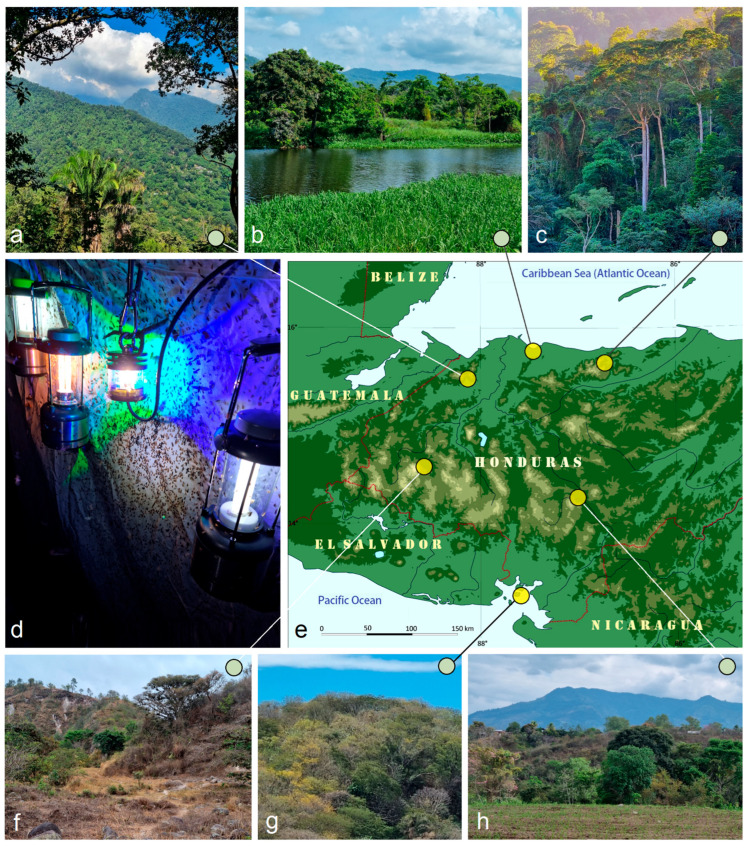
Study localities and sampling in Honduras: (**a**) El Merendon, suburbs of San Pedro Sula; (**b**) Tela, Caribbean coast in the Atlántida Department; (**c**) Pico Bonito, SE of La Ceiba, the right bank of the Río Cangrejal; (**d**) LepiLED lamp and fluorescent lanterns; (**e**) a map of the study localities; (**f**) Gracias, mountainous valley in the Lempira Department; (**g**) Isla Tigre, Gulf of Fonseca, Valle Department; (**h**) Cantarranas (=San Juan de Flores), the Río Grande o Choluteca valley, Francisco Morazán Department.

***Study Area and Seasonality.*** Field research in Honduras was conducted at six localities across five departments, each separated by approximately 100–180 km; the most distant sites—on the Caribbean and Pacific coasts—are about 280 km apart (Figure 1). These localities represent distinct ecoregions characterized by contrasting climatic conditions, ranging from tropical dry forests to tropical humid forests, although terminology varies among sources. For consistency with previous works (e.g., [22,23,24]), we follow the most comprehensive and recent ecoregional classification proposed by the Hierarchy Revisions Working Group, U.S. Department of Agriculture, Forest Service [25,26].

All fieldwork in Honduras was carried out during the dry season (February–April) in 2023, 2024, and 2025. This period was selected because adults of most Nepticulidae and Opostegidae species are most active at this time, and stable weather conditions ensure comparable sampling efficiency among sites and years. This seasonal window also provides optimal conditions for light-trapping, with minimal rainfall affecting nocturnal sampling.

***Sampling Methods.*** Consistent light-trapping and collecting procedures were applied at all Honduran localities to ensure comparable sampling effort and data quality. Where mains electricity was accessible, moths were attracted to a white vertical sheet illuminated by a Philips ML 160 W (220–230 V) mercury vapor bulb, suspended in a stable position to ensure optimal light dispersion. In remote areas without access to electricity, we employed the LepiLED lamp, a lightweight, compact device engineered for nocturnal Lepidoptera sampling [27], in combination with fluorescent lanterns powered by D-cell batteries [22,23,24] (Figure 1d).

**Figure 2:**
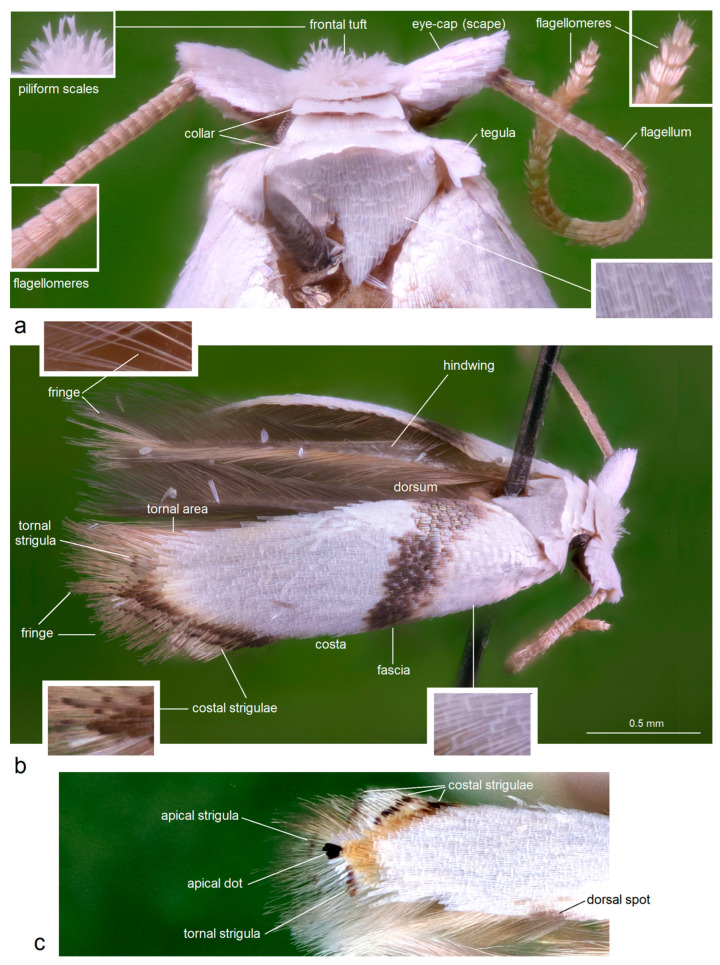
Adult morphology: (**a**) head and thorax, dorsal view, *Pseudopostega cristagalli* Stonis & Remeikis, sp. nov.; (**b**) same, dorso-lateral view of the adult; (**c**) apical half of the forewing, *P. mexicana* Remeikis & Stonis.

***Specimen Dissection and Documentation.*** Regarding morphological terminology, the descriptions of both adults (Figure 2) and genital structures follow the nomenclature established in the monographs by Puplesis and Robinson [9], Davis and Stonis [10], with only minor adjustments where necessary.

All procedures for specimen dissection, species identification, and morphological documentation followed protocols established in prior studies [9,10,12,17], with minor modifications adapted to this specific material. Male genitalia terminology follows the taxonomic revision by Donald R. Davis and J. R. Stonis [10], except for the term “socii” (a pair of setigerous lobes of the uncus), which is referred to here as “uncus”—a widely accepted usage [2] that has also been adopted in our previous publications on Opostegidae [9,12,17].

Male genitalia were dissected following standard techniques: abdomens were first macerated in a 10% potassium hydroxide (KOH) solution to soften and clear internal tissues, then thoroughly rinsed, cleaned, and dissected. Genital capsules were mounted on microscope slides with the ventral side oriented upward for consistent comparative analysis. In numerous cases, the phallus was carefully separated from the capsule and mounted under a separate cover slip on the same slide to facilitate clearer imaging and structural interpretation. Abdominal pelts were not retained for this study. Permanent slide mounts were photographed and studied using a Leica DM2500 compound microscope equipped with a Leica DFC420 digital camera (Leica company, Wetzlar, Germany), allowing for high-resolution imaging of genital structures. Adult moths were examined and measured under a Lomo MBS-10 stereomicroscope (Lomo company, St. Petersburg, Russia), while additional imaging was performed using a Leica S6D stereomicroscope paired with a Leica DFC290 digital camera (Leica company, Wetzlar, Germany). In imaging male genitalia, we deliberately avoided image stacking (e.g., using Leica’s “Multifocus” function). For these specimens, the strong overlap of structures made stacked images problematic: such processing obscured structural boundaries and made it difficult to determine how certain parts were connected or to which anatomical layer (dorsal or ventral) they belonged. Genitalia measurements were rounded off to the nearest 5 μm.

***Molecular Analysis.*** Total genomic DNA was extracted from legs or whole air-dried adults with the GeneJet Genomic DNA Purification Kit (Thermo Fisher Scientific Baltics, Vilnius, Lithuania). Amplification of the partial cytochrome c oxidase subunit 1 of the mitochondrial DNA (mtDNA CO1-5′) was performed with LCO1490 and HCO2198 primers [28,29]. PCR mixture involved 12.5 µL of 2× DreamTaq PCR Master Mix (Thermo Fisher Scientific Baltics, Vilnius, Lithuania), 2.5 µL of each primer (Metabion, Planegg, Germany), 2 µL of genomic DNA, and 5.5 µL deionised H_2_O. PCR conditions were as follows: initial denaturation at 95 °C for 5 min; 45 cycles of denaturation at 94 °C for 40 s, annealing at 45 °C for 40 s, synthesis at 72 °C for 1 min; final synthesis at 72 °C for 5 min. Successfully amplified PCR products were purified with exonuclease I and FastAP thermosensitive alkaline phosphatase (Thermo Fisher Scientific Baltics, Vilnius, Lithuania) and sent to Macrogen Europe (Amsterdam, The Netherlands) for Sanger sequencing.

Obtained sequences were manually aligned using BioEdit v.7.2.5 [30] and deposited in the National Genomics Data Center (NGDC, China) [31] under the accession IDs SAMC6087948–SAMC6087980. Additionally, sequences previously published by the authors [19,23,32] and downloaded from the BOLD platform [33] were used.

Neighbor-Joining (NJ) (TN93 + G model, 10,000 bootstrap replicates) and Maximum Likelihood (ML) (GTR + G + I model, 10,000 replicates) trees were constructed using MEGA v.7 [34]. Bayesian inference (GTR + G + I, 10 million generations) was applied using the MrBayes v.3.2.3 program [35] and visualised with FigTree v.1.4.4 [36]. Species delimitation was performed using the bPTP method [37]. The mitotype network was constructed using TCS Network [38] implemented in PopArt v.1.7 [39].

While analyzing sequences obtained from public databases (GenBank and BOLD), we carefully evaluated their taxonomic accuracy by comparing phylogenetic placement and morphological evidence. Sequences labeled under the same species name but forming distinctly separate clusters in our molecular phylogenies, and grouping with unrelated species, were regarded as doubtful or potentially misidentified.

Additionally, sequences listed in GenBank or BOLD under one species name (e.g., “species X”) but showing full or near-identical matches with our own sequences of another, morphologically confirmed species (e.g., “species Y”) were also treated as *misidentified*. These cases were cross-checked through morphological examination of voucher material, whenever available, to ensure consistency between molecular and morphological data.

***Abbreviations for Institutions and Specimen Depositories.*** BRG—Biosystematics Research Group, currently based at the NRC, Vilnius, Lithuania; MfN—Museum für Naturkunde, formerly known as the Museum für Naturkunde der Humboldt-Universität zu Berlin, Germany; NHMUK (formerly BMNH)—the Natural History Museum, London, United Kingdom; NRC—State Scientific Research Institute Nature Research Centre, Vilnius, Lithuania; PUJ—Departamento de Biología, Pontificia Universidad Javeriana (Xavierian Pontifical University), Bogotá, Colombia.

## 3. Results

### 3.1. Re-Examination of Historical Material from the Collection of the NHMUK (London, UK)

#### 3.1.1. First Documentation of the True *Pseudopostega saltatrix* (Walsingham, 1897)

**Figure 3:**
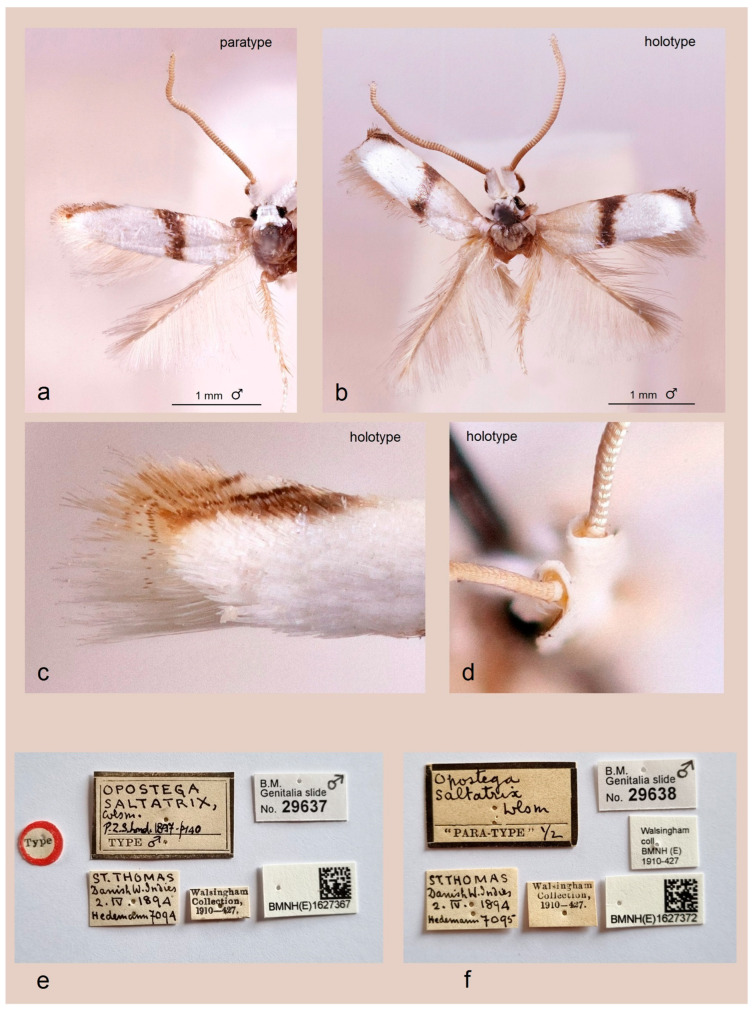
*Pseudopostega saltatrix* (Walsingham), type series, historical material deposited in the NHMUK collection: (**a**) male paratype; (**b**–**d**) male holotype; (**e**,**f**) specimen labels.

The type series—and especially the holotype—is crucial for accurately identifying a species. *Pseudopostega saltatrix* was described by Thomas de Grey Walsingham, and its type series is deposited in the collection of the Natural History Museum, London (NHMUK) (Figure 3).

*Material examined*. 1 ♂, holotype, Danish West Indies (currently U.S. Virgin Islands), St. Thomas, 2 April 1894, leg. Hedemann, no. 7094, T.G. Walsingham Collection, no. 1910-427, genitalia slide no. BMNH29637 (01627367 NHMUK). 1 ♂, paratype, same locality and date as holotype, leg. Hedemann, no. 7095, T.G. Walsingham Collection, no. 1910-427, genitalia slide no. BMNH29638 (01627372 NHMUK).

For the very first time, the *Pseudopostega saltatrix* (Walsingham) specimens preserved at the NHMUK were dissected by Arūnas Diškus (BRG) and subsequently studied by the first author of the present article in December 2001. The British material of *P. saltatrix* comprises two specimens from the T.G. Walsingham Collection: a male holotype (genitalia slide no. BMNH 29637) and one male paratype (genitalia slide no. BMNH 29638) (both collected in 1894 on St. Thomas Island, Danish West Indies, currently the U.S. Virgin Islands).

First of all, our re-examination showed that both specimens of the type series are completely identical in terms of external characteristics as well as male genital structures.

In adults (Figure 3a–d), the characteristic dark transverse oblique fascia is submedian, i.e., it originates clearly distant from the wing base (not reaching the thorax when the moth is at rest with wings closed). Notably, the tegulae are dark brown proximally. The apical strigulae of the forewing consist of three costal strigulae and one tornal strigula, and there is no distinct black apical dot; instead, there is an irregular brown apical spot, which is most likely somewhat faded in these old collection specimens. The forewing length ranges from 2.1 to 2.2 mm; the wingspan ranges from 4.9 to 5.1 mm.

**Figure 4:**
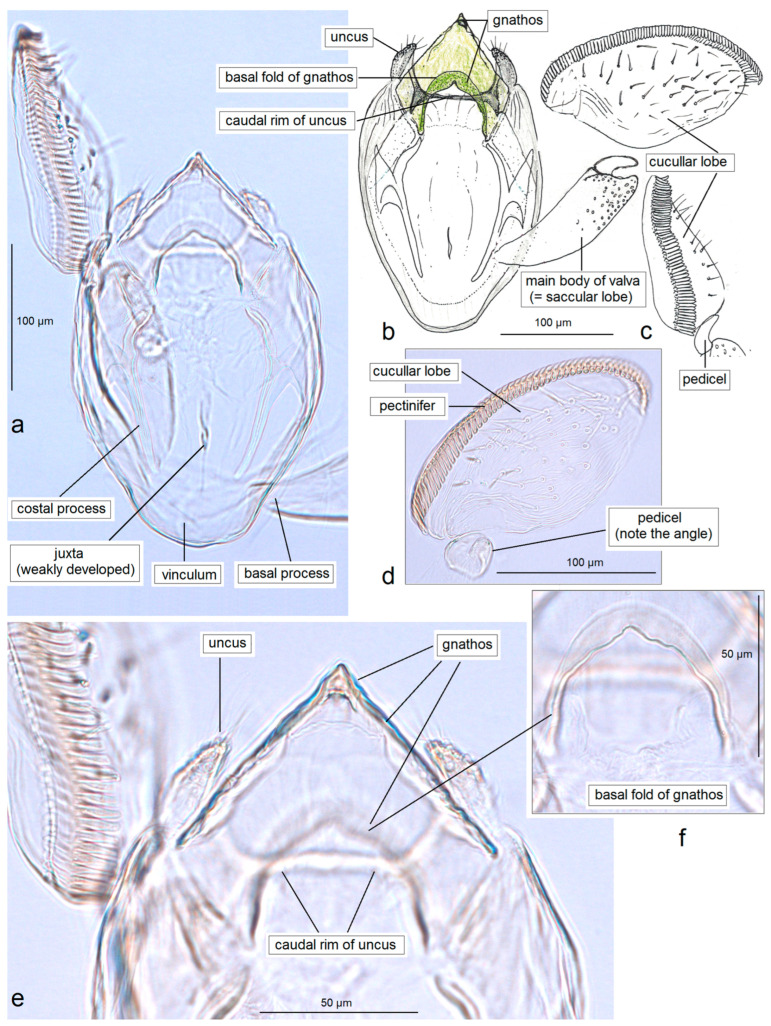
Male genitalia of *Pseudopostega saltatrix* (Walsingham), holotype, genitalia slide no. BMNH29637 (01627367 NHMUK): (**a**,**b**) general view; (**c**,**d**) cucullar lobe; (**e**,**f**) uncus and gnathos.

**Figure 5:**
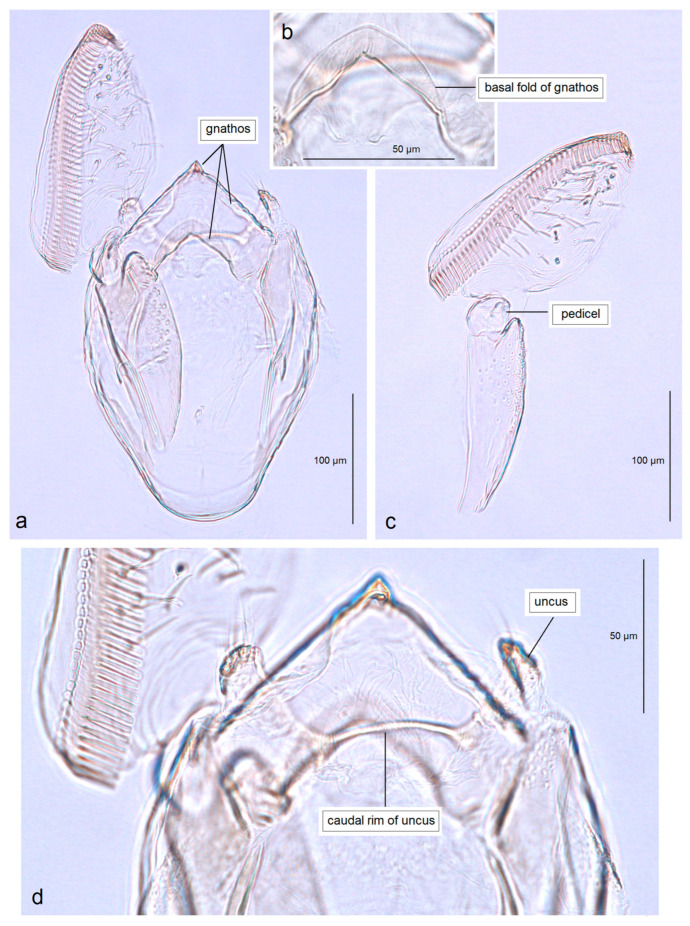
Male genitalia of *Pseudopostega saltatrix* (Walsingham), paratype, genitalia slide no. BMNH29638 (01627372 NHMUK): (**a**) general view; (**b**) basal fold of the gnathos; (**c**) valva; (**d**) uncus.

In addition, the examination of the male genitalia (Figure 4 and Figure 5) revealed that this species is characterized by a long caudal rim of the uncus with laterally separated uncus lobes. The gnathos is triangular, with a ventrally curved, hook-shaped apex, but lacks comb-like papillae or any other distinctly chitinized structures in the median part, as seen in the gnathos of another, closely related, newly collected species from Honduras. The basal fold of the gnathos lacks a chitinized lobe-like anterior projection and is membranous anteriorly.

In the revision of New World Opostegidae [10], the available unfinished sketch of the male genitalia of the *P. saltatrix* holotype was unfortunately neglected and remained unpublished. For the purpose of illustrating this species, the authors instead selected an already completed Indian ink drawing of a misidentified Jamaican specimen (see Davis and Stonis [10]: fig. 336). Moreover, following erroneous assumptions about *P. saltatrix* being a highly variable species, the revision presented numerous adult illustrations drawn by J. R. Stonis under the name “*saltatrix*” (figs. 183–190 [10]), of which only one (fig. 186) actually depicts the adult holotype of the true *P. saltatrix*. The remaining illustrations represent a mixture of species, potentially including undescribed and unnamed taxa. Our current re-examination of the type material of *P. saltatrix* from the T.G. Walsingham Collection (NHMUK) indicates that the male genitalia of the true *P. saltatrix* differ from those of the “*P. saltatrix*” presented by the revision of New World Opostegidae [10]. Therefore, based on our re-examination of slide nos. BMNH 29637 and 29638, we provide the first formal documentation of *P. saltatrix*. Currently, there are no molecular data available for *P. saltatrix*; the only published DNA sequences attributed to *P. saltatrix*, based on specimens from Costa Rica, belong to another, new species (not *P. saltatrix*) (see *Molecular Considerations*).

Knowing that the *P. saltatrix* group includes many similar but distinct species, during our study of historical material at the NHMUK, the Jamaican female specimen (Runaway Bay, Jamaica, 28 February 1905, T.G. Walsingham Collection, no. 78259, genitalia slide no. BMNH29640)—illustrated in detail in fig. 459 [10]—was treated by us only as “a possible *P. saltatrix*”. Despite the external similarity of this female to specimens from the type series of *P. saltatrix* and the relatively close locality of collection, doubts remain whether this female truly belongs to *P. saltatrix* or to another species within the speciose *saltatrix* group (e.g., *P. jamaicensis* Stonis & Remeikis, sp. nov., described below).

*Remark:* None of the American specimens listed or illustrated in the monograph by Davis and Stonis [10] are currently accessible to us.

#### 3.1.2. Description of *Pseudopostega jamaicensis* Stonis & Remeikis, sp. nov., a New Species Related to *P. saltatrix* (Walsingham)

**Figure 6:**
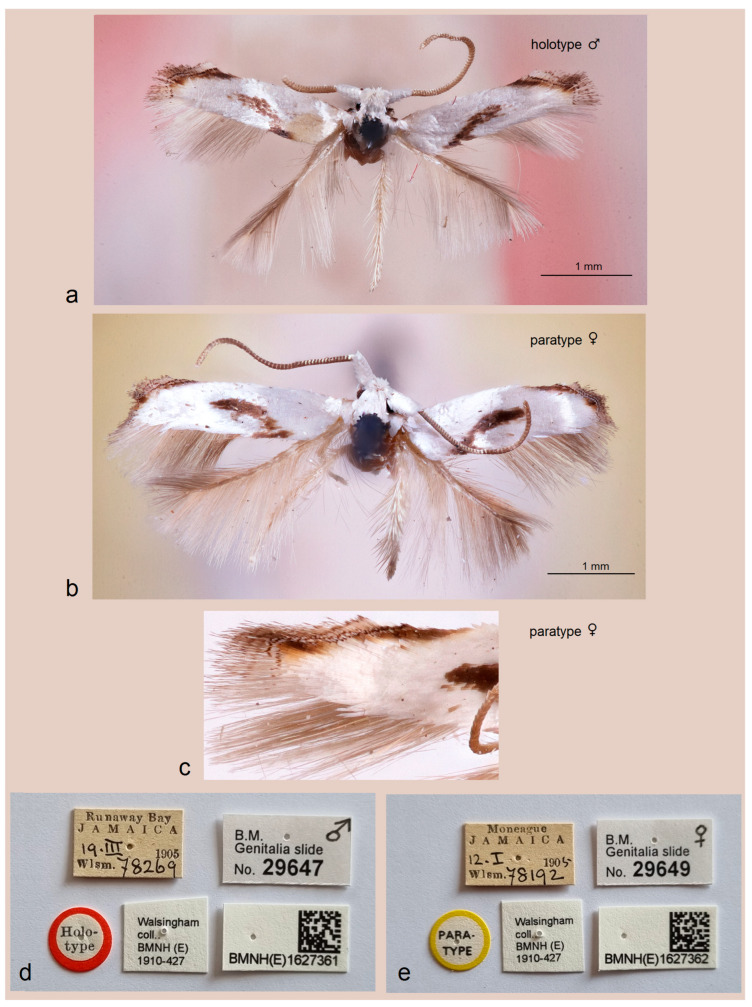
*Pseudopostega jamaicensis* Stonis & Remeikis, sp. nov., historical material deposited in the NHMUK collection: (**a**) male holotype; (**b**,**c**) female paratype, (**d**,**e**) labels.

During the re-examination of historical material from the NHMUK collection, we detected a new species (Figure 6), which is described below.

*ZooBank Registration*. https://zoobank.org/NomenclaturalActs/8ae67c57-cc33-4ba5-8afc-6d472dd19d5e (accessed on 28 October 2025).

*Type material.* Holotype, ♂, Jamaica, Runaway Bay, 19 March 1905, T.G. Walsingham Collection, no. 78269, genitalia slide no. BMNH29647 (NHMUK). Paratype: 1 ♀, same locality as holotype, 12 January 1905, T.G. Walsingham Collection, no. 78192, genitalia slide no. BMNH29649 (NHMUK).

*Diagnosis.* The new species differs from other *Pseudopostega*, including *P. saltatrix* (Walsingham), due to the combination of a distinctive, elongated, oblique, fascia-like dorsal spot on the forewing, slender male genital capsule, and a simply shaped gnathos with square lateral thickenings.

*Barcode.* There are no DNA sequences available for this species.

*Male* (Figure 6a). The forewing length is 2.2 mm; the wingspan is 5.1 mm. Head: the palpi are ochreous cream; the frontal tuft and collar are snow white and glossy; the antenna is nearly as long as the forewing; the flagellum is ochreous cream and comprises about 62 flagellomeres. Thorax: the tegulae and the thorax are snow white; the forewing is snow white with a large, elongated tornal oblique band of dark brown scales; the apex of the forewing has three costal, one apical, and two tornal strigulae; the fringe is brown on the costal margin, otherwise it is cream white; the forewing underside is brown; hindwing is brownish grey, with some white scales at the extended base; the fringe is brownish cream; the legs are glossy ochreous cream. Abdomen: the abdomen is ochreous brown dorsally and cream white ventrally.

*Female* (Figure 6b,c). The forewing length is 2.4 mm; the wingspan is 5.7 mm. Otherwise, it is similar to the male.

**Figure 7:**
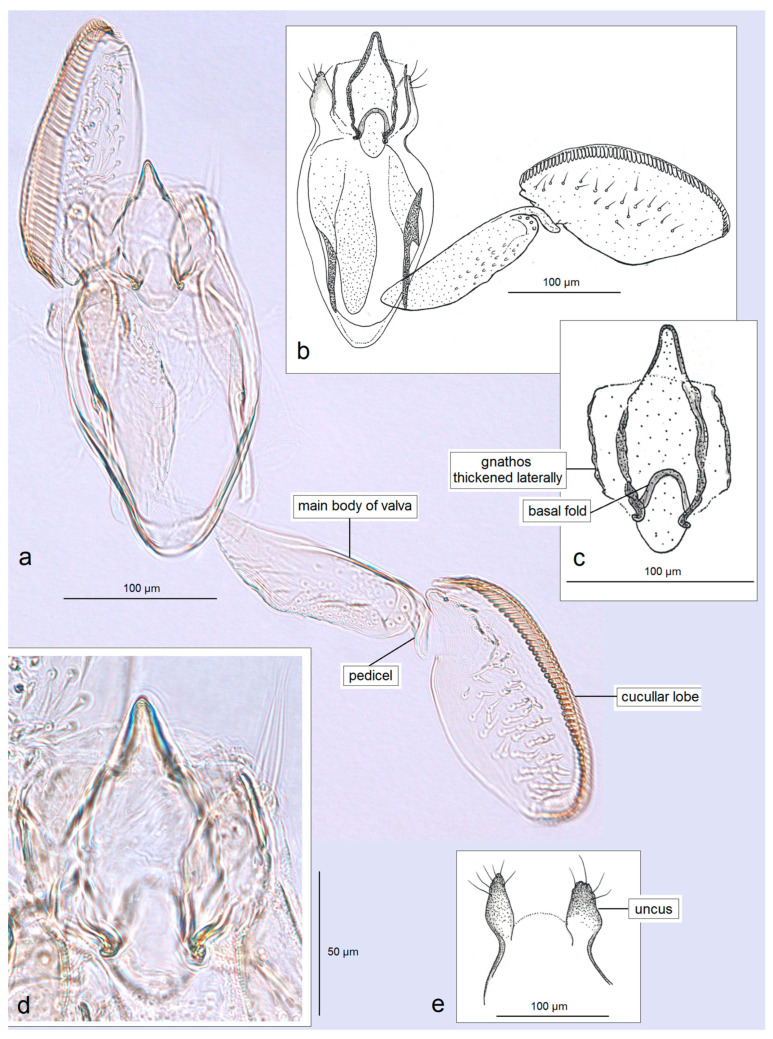
Male genitalia of *Pseudopostega jamaicensis* Stonis & Remeikis, sp. nov., holotype, genitalia slide no. BMNH29647 (NHMUK): (**a**,**b**) general view; (**c**,**d**) gnathos; (**e**) uncus.

*Male genitalia* (Figure 7). The capsule is slender, measuring 265 µm long and 130 µm wide. The uncus has two large, elongated lateral lobes. The gnathos has a simple-shaped, distally triangular median element, a small rounded basal fold, and square-like lateral thickenings. The valva is about 345 µm long: the main body (saccular lobe) is 165 µm long, and the cucullar lobe is 180 µm long and 90 µm wide. The transtilla is absent. The vinculum is slender, distally rounded, and without lateral lobes. The juxta is absent.

**Figure 8:**
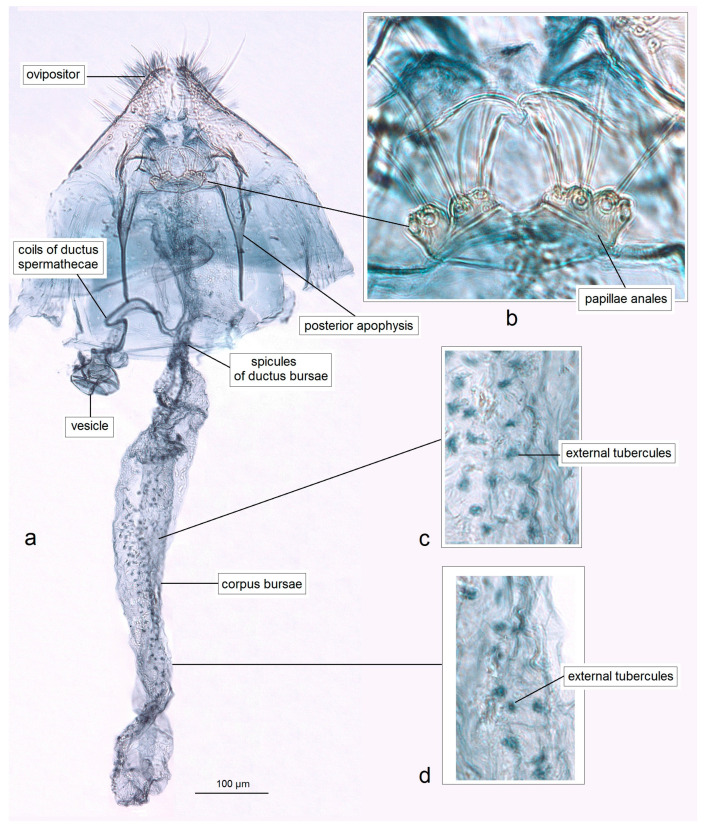
Female genitalia of *Pseudopostega jamaicensis* Stonis & Remeikis, sp. nov., paratype, genitalia slide no. BMNH29649 (NHMUK): (**a**) general view; (**b**) enlarged view of papillae anales; (**c**,**d**) fragments of corpus bursae.

*Female genitalia* (Figure 8). The total length is 1085 µm. The ovipositor is short, triangular, and two-folded. The posterior apophyses are slender and about 175 µm long. The papillae anales are distinctly bilobed. The ductus bursae possesses tiny spicules. The ductus spermathecae has some coils and a large, complex vesicle. The corpus bursae is characterized by a band of external tubercles.

*Bionomics*. Adults fly in January–March; otherwise, the biology of this species is unknown.

*Distribution*. Currently, the species is only known from Jamaica.

*Etymology.* The species name is derived from Jamaica, the country where the type specimens were collected in 1905, one hundred and twenty years ago.

*Remarks*. The description of the new species is based on two specimens: a male holotype and a female paratype. It is possible that a few more specimens of *P. jamaicensis* sp. nov. can be found in the collections of the Smithsonian Institution (USA) on unreturned loan from NHMUK or other Latin American institutions. However, this material (if any) is not currently accessible.

### 3.2. The First Attempt to Assess the Taxonomic Diversity of Opostegidae in Honduras, Resulting in the Discovery of New Species

**Figure 9:**
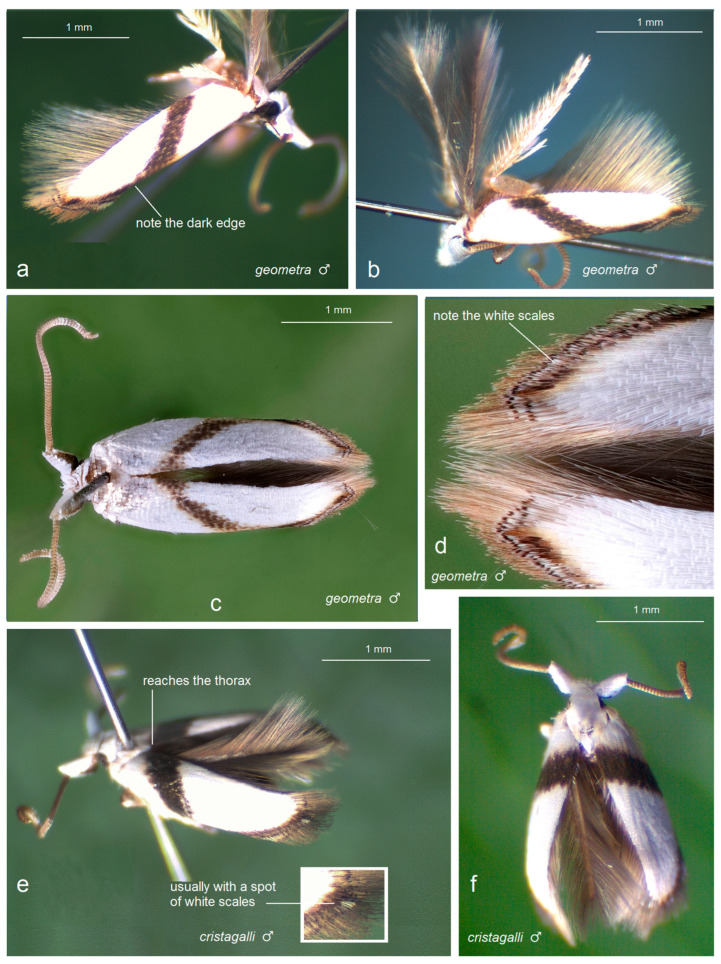
Adults of *Pseudopostega* Kozlov species from Honduras: (**a**) *P. geometra* Stonis & Remeikis, sp. nov., Cantarranas, with the genitalia slide label no. RA1257; (**b**) same, Pico Bonito, with the genitalia slide label no. RA1278; (**c**,**d**) same, with the genitalia slide label no. RA1259; (**e**,**f**) *P. cristagalli* Stonis & Remeikis, sp. nov., Tela, 19 March 2024, without genitalia slide.

**Figure 10:**
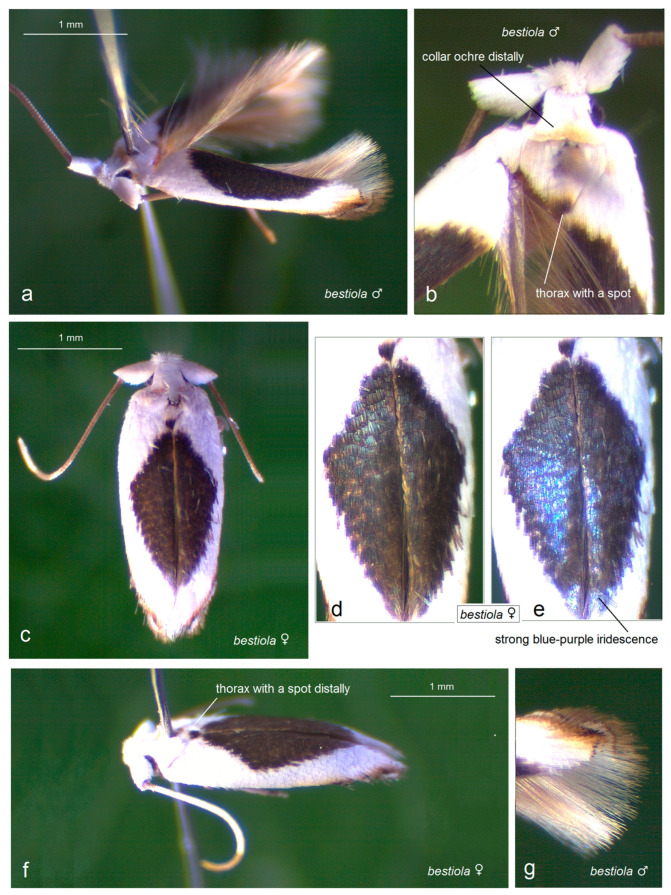
Adults of *Pseudopostega bestiola* Stonis & Remeikis, sp. nov.: (**a**,**b**) Cantarranas, 18 April 2023; (**c**–**f**) same, 10 February 2024; (**g**) same, 18 April 2023.

**Figure 11:**
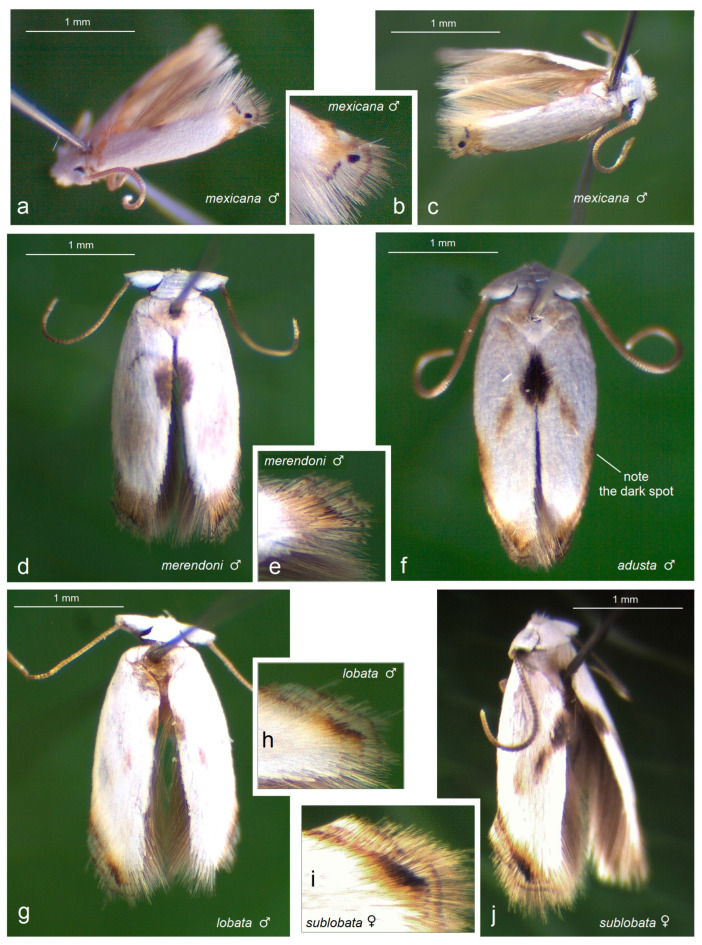
Adults of *Pseudopostega* Kozlov species from Honduras: (**a**–**c**) *P. mexicana* Remeikis & Stonis, Isla Tigre, 18 February 2023; (**d**,**e**) *P. merendoni* Stonis & Remeikis, sp. nov., San Pedro Sula (Merendon), with the genitalia slide label no. RA1276; (**f**) *P. adusta* (Walsingham), 2 April 2024, without genitalia slide; (**g**,**h**) *P. lobata* Davis & Stonis, with the genitalia slide label no. RA1275; (**i**,**j**) *P. sublobata* Davis & Stonis, San Pedro Sula (El Merendon), 11 February 2023, without genitalia slide.

**Figure 12:**
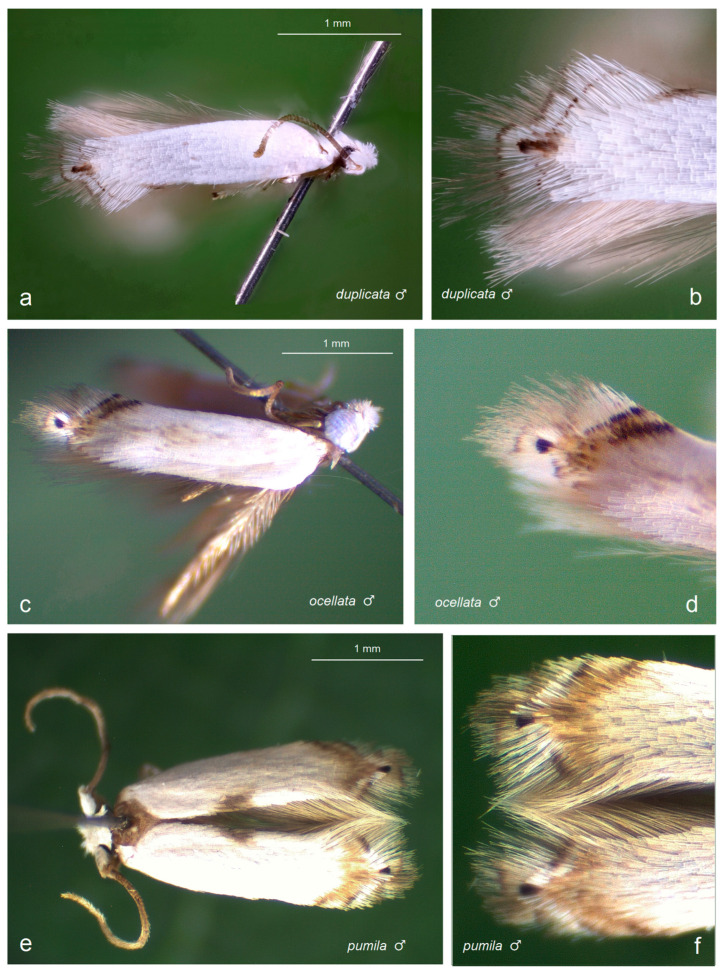
Adults of *Pseudopostega* Kozlov species from Honduras: (**a**,**b**) *P. duplicata* Davis & Stonis, Cantarranas, with the genitalia slide label no. RA1273; (**c**,**d**) *P. ocellata* Stonis & Remeikis, sp. nov. with genitalia slide label no. RA1264; (**e**,**f**) *P. pumila* (Walsingham), Tela, 27 February 2024.

Prior to our research, no studies on Opostegidae had ever been conducted in Honduras. Based on material sampled from five distinct localities across Honduras, dissection and taxonomic identification of the specimens—primarily through the study of male genitalia and external morphological characters—revealed a total of 11 species of *Pseudopostega* Kozlov (Figure 9, Figure 10, Figure 11 and Figure 12). These include six previously described species that are here recorded for the first time in Honduras: *Pseudopostega mexicana* Remeikis & Stonis, formerly known from the Pacific coast of Mexico [21]; *P. adusta* (Walsingham), a widespread species ranging from the Caribbean to the Amazon region of Ecuador [10]; *P. lobata* Davis & Stonis, previously reported from Central America and northwestern Argentina [10] and Ecuador [21]; *P. sublobata* Davis & Stonis, known from Costa Rica and Ecuador [10], as well as Peru [19]; *P. duplicata* Davis & Stonis, previously known from Costa Rica and the British Virgin Islands [10]; and *P. pumila* (Walsingham), earlier recorded from tropical Mexico [10]. Since no species of Opostegidae had been reported from Honduras until now, all of these represent new national records. In addition, the material yielded five species new to science: *Pseudopostega geometra* Stonis & Remeikis, sp. nov., *P. cristagalli* Stonis & Remeikis, sp. nov., *P. bestiola* Stonis & Remeikis, sp. nov., *P. merendoni* Stonis & Remeikis, sp. nov., and *P. ocellata* Stonis & Remeikis, sp. nov. These new species are described below in a detailed taxonomic account of the findings from Honduras.

#### 3.2.1. *Pseudopostega geometra* Stonis & Remeikis, sp. nov.

*ZooBank Registration*. https://zoobank.org/NomenclaturalActs/a16d93a2-c835-4359-9804-c09cb15f2dcf (accessed on 28 October 2025).

*Diagnosis.* The new species, *Pseudopostega geometra* sp. nov., is assigned to the *P. saltatrix* species group. Externally, this new species is characterized by the distinct, apically angular forewing pattern in which the oblique dark fascia is coalescent with the costal strigulae; the latter are triangularly curved. In the male genitalia, *P. geometra* is distinguished from all other *Pseudopostega* species, including those of the *P. saltatrix* group, by the unique gnathos with a bullet-shaped caudal process.

*Barcodes.* We barcoded five male specimens: one paratype from Tela, two paratypes from La Ceiba (Pico Bonito), one paratype from Cantarranas, and one non-type specimen from Tegucigalpa, Honduras. Their sequences have been deposited in the National Genomics Data Center (NGDC, China) (accession IDs: SAMC6087948– SAMC6087952).

*Male* (Figure 9a–d). The forewing ranges from 1.9 to 2.7 mm; the wingspan ranges from 4.4 to 6 mm (*n* = 5). The frontal tuft is short, snow white. The collar and the thorax are glossy snow white. The forewing is glossy snow white, with oblique brown-black to brown fascia, which is connected with apical strigulae. The apical strigulae are triangularly curved, formed by black-brown or dark brown scales, with indistinctive pale yellowish ochre shadow anteriorly; the apical dot is black, elongated, sometimes weakly defined; the area between two strigulae is white. The fringe varies from pale brown to dark brown. The hindwing is brown; its fringe varies from pale brown to ochre cream.

*Female*. The female of this species remains unknown.

**Figure 13:**
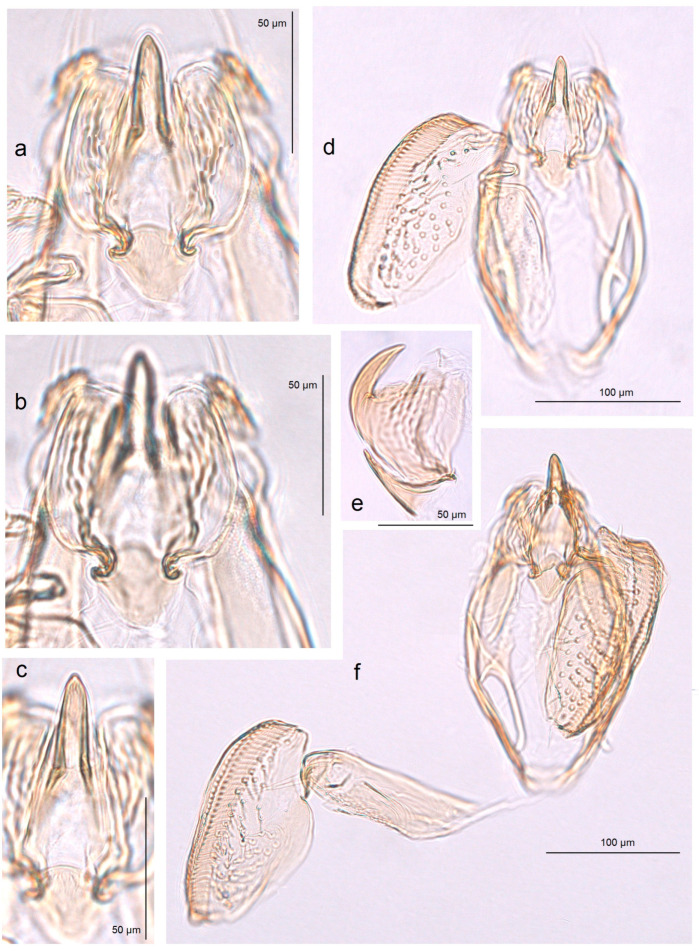
Male genitalia of *Pseudopostega geometra* Stonis & Remeikis, sp. nov.: (**a**–**c**), gnathos ventrally, paratype, slide no. RA1257; (**d**) same, general view; (**e**) gnathos laterally, paratype, slide no. RA1281; (**f**) general view, holotype, slide no. RA1278 (MfN).

**Figure 14:**
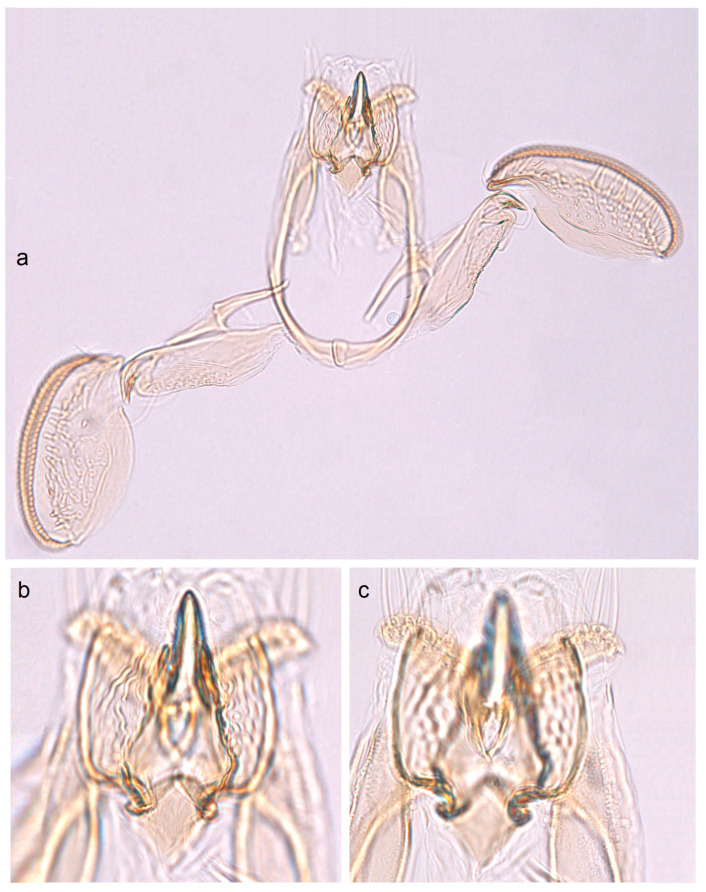
Male genitalia of *Pseudopostega geometra* Stonis & Remeikis, sp. nov.: (**a**) general view, aberrant paratype specimen, genitalia slide no. RA1279 (MfN); (**b**,**c**) same, gnathos and uncus.

*Male genitalia* (Figure 13 and Figure 14). The capsule, measuring 225–270 µm in length and 120–140 µm in width, has an uncus with two large triangular lateral lobes. The gnathos has wrinkled angular lobes laterally, a small, hood-like basal fold, and a specific, bullet-shaped caudal process; the latter, when viewed in lateral view, is curved ventrally. The valva bears a large cucullar lobe, measuring 150–180 µm in length and 70–90 µm in width. The vinculum is tapering, without lateral lobes distally; in the aberrant specimen (slide no. RA1279) is rounded and thickened distally.

*Bionomics*. Adults are active from February to April and fly towards light. Otherwise, the species’ biology is unknown.

*Distribution*. The new species is known from various localities in Honduras: from the Caribbean (Atlantic) coast to mountainous valleys in the western and central parts of the country, at elevations ranging from 10 m (Tela) to 1100 m (Tegucigalpa). According to molecular sequences, the species also occurs in Costa Rica and Mexico (see *Molecular Considerations*).

*Material examined*. Holotype: ♂, Honduras, Atlántida Department, 7.5 km south east of La Ceiba, Villas Pico Bonito, 100 m, 15°43′39″N, 86°44′31″W, 16 April 2023, leg. J.R. Stonis, genitalia slide no. RA1278♂ (MfN). Paratypes: 1 ♂, the same label data as the holotype, genitalia slide no. RA1281♂ (no pinned specimen was preserved; it was taken for DNA extraction) (MfN); 1 ♂, 7.5 km south east of La Ceiba, the right bank of the Río Cangrejal, Hotel Rio, 100 m, 15°43′34″N, 86°44′26″W, 14 April 2023, leg. J.R. Stonis, genitalia slide no. RA 1277♂ (no pinned specimen was preserved; it was taken for DNA extraction) (MfN); 1 ♂, Atlántida Department, Tela, 10–30 m, 15°45′56″N, 87°27′06″W–15°46′09″N, 87°27′15″W, 2–19 April 2024, leg. J.R. Stonis, genitalia slide no. RA1280♂ (no pinned specimen was preserved; it was taken for DNA extraction) (MfN); 1 ♂, same label data as the previous, genitalia slide no. RA1279♂ (aberrant) (MfN); 1 ♂, Francisco Morazán Department, Cantarranas (=San Juan de Flores), 660–690 m, 14°16′17″N, 87°01′07″W–14°15′50″N, 87°01′35″W, 10–15 February 2024, genitalia slide no. RA1257 (MfN); 1 ♂, Lempira Department, Gracias, via Gualtepeque, 750 m, 14°35′35″N, 88°34′36″W, 28–31 March 2024, genitalia slide no. RA1259 (MfN). Also see *Remark*.

*Etymology*. The species name refers to the geometrical pattern of the forewing, characterized by the sharply angular and linear arrangement of the apical strigulae. It is treated as a Latin feminine noun in apposition.

*Remark.* One female specimen was collected in Francisco Morazán Department, Tegucigalpa, Picacho, 26 April 2024, leg. J.R. Stonis; it was used for DNA extraction (sequence no. SAMC6087952), and neither genitalia nor pinned adult is available.

#### 3.2.2. *Pseudopostega cristagalli* Stonis & Remeikis, sp. nov.

*ZooBank Registration*. https://zoobank.org/NomenclaturalActs/4e41bb25-4a14-4d1a-ba6f-be1fd0fb7125 (accessed on 28 October 2025).

*Diagnosis*. The new species, *Pseudopostega cristagalli*, sp. nov., is assigned to the *P. saltatrix* species group. Externally, this new species is easily distinguished by the distinct forewing pattern in which the oblique dark fascia reaches or almost reaches the thorax dorsally. In the male genitalia, *P. cristagalli* is distinguished from all other *Pseudopostega* species, including those of the *P. saltatrix* group, by the unique gnathos possessing small, comb-like papillae near the stout caudal process (positioned laterally) and the rounded genital capsule.

*Barcodes*. We barcoded seven non-type series specimens of both sexes collected at the same locality in Tela, Atlántida Department, at the same time as the specimens of the type series: on 12 March–9 April and 13–19 April 2024. Their sequences have been deposited in the National Genomics Data Center (NGDC, China) (accession IDs: SAMC6087953–SAMC6087959).

*Male* (Figure 2a,b and Figure 9e,f). The forewing ranges from 2.0 to 2.1 mm; the wingspan ranges from 4.5 to 4.7 mm (*n* = 11). The frontal tuft is short, snow white. The collar and the thorax are glossy snow white. The forewing is glossy snow white, with oblique dark fascia which varies in colour from golden glossy dark grey-brown to black-brown with golden gloss; the fascia reaches or nearly reaches the thorax dorsally. The first and the second costal strigulae are from dark brown to black-brown; the third strigula is pale brown, sometimes weakly defined; the area proximal to the costal strigulae is slightly shadowed with a yellowish tint; the apical dot is black, usually triangular and almost always parallel with a distinctive (and diagnostic) small spot of white scales. The fringe varies from brown to dark brown. The underside of the forewing is brown-black. The hindwing and its fringe are brown. The legs are glossy ochre cream, with some brown-black scales on the upper side distally; the forelegs are almost entirely covered with brown-black scales on the upper side. The abdomen is brown-black on the upper side and glossy cream on the underside; the anal tufts are medium short, cream or ochre cream, comprising widened piliform scales.

*Female.* The forewing ranges from 2.3 to 2.4 mm; the wingspan ranges from 5.2 to 5.5 mm (*n* = 5). The scaling of the female is similar to those of the male.

**Figure 15:**
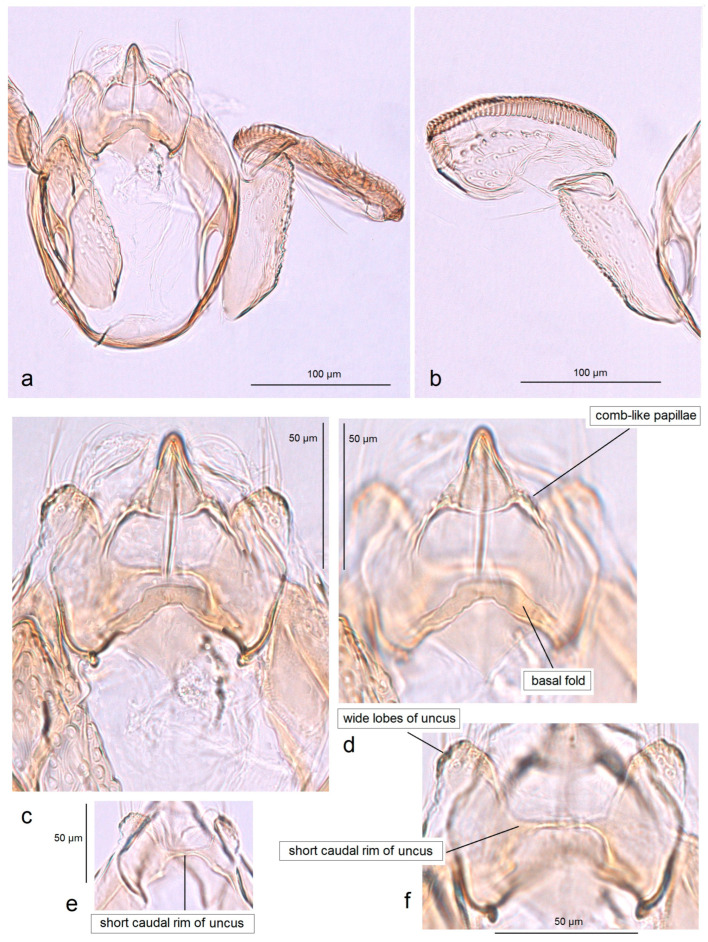
Male genitalia of *Pseudopostega cristagalli* Stonis & Remeikis, sp. nov.: (**a**,**c**,**d**,**f**) holotype, genitalia slide no. RA1268; (**b**,**e**) paratype, genitalia slide RA1261 (MfN).

*Male genitalia* (Figure 15). The capsule is rounded; it ranges from 220 to 230 µm in length and from 150 to 160 µm in width. The uncus possesses two large, well-separated, distally rounded lateral lobes. The gnathos has smooth, laterally thickened lateral lobes, a medium-large, hood-like basal fold, and a specific, stout caudal process with small, unique comb-like papillae laterally. The valva bears a large cucullar lobe which ranges from 130 to 140 µm in length, and from 70 to 75 µm in width. The vinculum is rounded distally, without lateral lobes. The juxta is absent.

**Figure 16:**
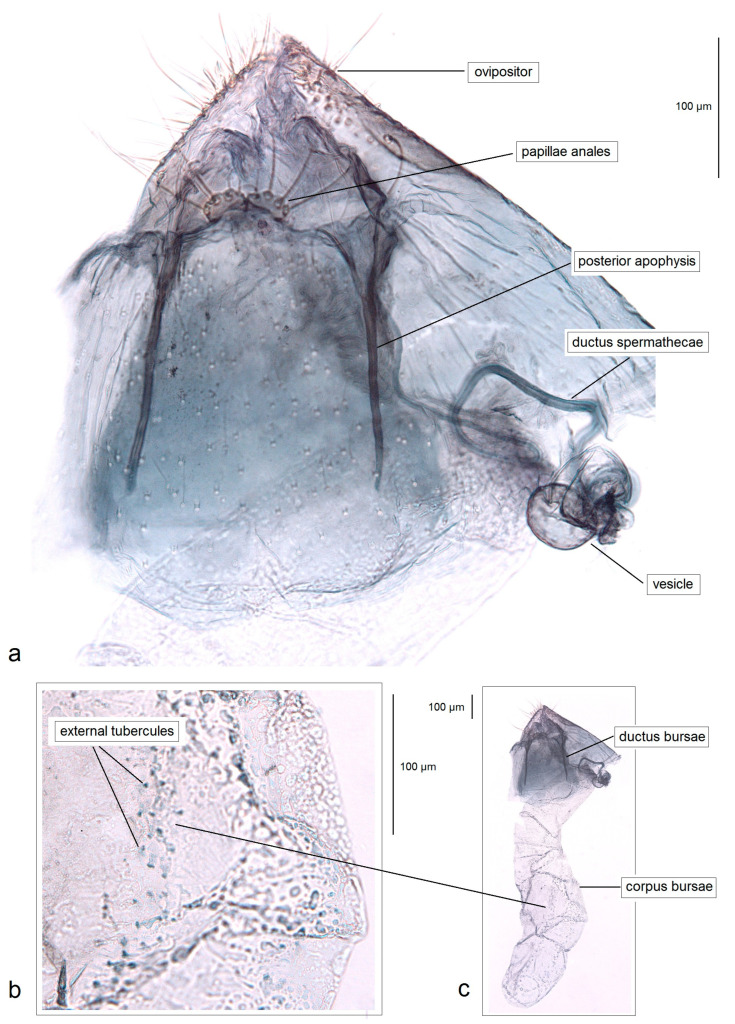
Female genitalia of *Pseudopostega cristagalli* Stonis & Remeikis, sp. nov., paratype, no. RA1282 (MfN): (**a**) ovipositor; (**b**) fragment of corpus bursae; (**c**) general view.

*Female genitalia* (Figure 16). The total length is about 1220 µm. The abdomen tapers to a triangularly shaped ovipositor. The posterior apophyses are slender. The anal papillae are bilobed. The vestibulum is wide and membranous. The ductus bursae is elongated, measuring 210–230 µm in length; it bears external tubercules arranged in a slender band. The ductus spermathecae is relatively short, with 1.5–2 coils and a rounded vesicle.

*Bionomics*. Adults are active from February to April and fly towards light. Otherwise, the species’ biology is unknown.

*Distribution*. The new species is known from only a single locality in Honduras: Tela, Atlántida Department, along the Caribbean coast, at elevations from 10 to 30 m.

*Material examined*. Holotype: ♂, Honduras, Atlántida Department, Tela, 10–20 m, 15°45′56″N, 87°27′06″W–15°46′35″N, 87°27′18″W, 27 February–19 March 2024, leg. J.R. Stonis, genitalia slide no. RA1268♂ (MfN). Paratypes: 6 ♂, 3 ♀, the same label data as the holotype, genitalia slide nos. RA1260♂, RA1261♂, RA1282♀ (MfN); 5 ♂, 2 ♀, the same locality, 2–19 April 2024, leg. J.R. Stonis, genitalia slide no. RA1287♂ (MfN).

*Etymology*. The species name *cristagalli* is a Latin genitive noun meaning “of the rooster’s comb” (*crista galli*), referring to the distinctive morphology of the gnathos in the male genitalia. This structure bears some resemblance to a rooster’s comb, making it a key diagnostic feature for recognizing this species among congeners.

#### 3.2.3. *Pseudopostega bestiola* Stonis & Remeikis, sp. nov.

*ZooBank Registration*. https://zoobank.org/NomenclaturalActs/7edf659c-7234-4054-90e4-9095cfca8a8c (accessed on 28 September 2025).

*Diagnosis*. The new species, *Pseudopostega bestiola* sp. nov., is currently assigned to the *P. latifurcata* species group. Externally, this new species is characterized by the distinct, rare pattern where the dark markings of the forewings form a massive spot when both wings are closed. Moreover, the new species possesses a unique distal dark spot on the thorax and an ochre shadow on the distal edge of the collar. A similar pattern, but without a spot on the thorax and the shadow on the collar, is also characteristic of *P. dorsalis* Davis & Stonis and *P. colognatha* Davis & Stonis; however, these two latter species possess very different male genitalia and belong to another species group, the *P. saltatrix* group. The Costa Rican *P. bidorsalis* Davis & Stonis seems to be the most similar and probably related; however, the presence of spines, the truncated processes of the gnathos in the male genitalia, and the pointed ovipositor in the female genitalia distinguish the new species from *P. bidorsalis*. The Caribbean *P. latifurcata* Davis & Stonis, a species also possessing a massive dorsal spot, clearly differs from the new species in the genitalia. In the male genitalia, *P. bestiola* sp. nov. is also similar to the Jamaican *P. longifurcata* and the Ecuadorian *P. spinosa* Stonis & Diškus (both of the *P. brachybasis* species group) but differs by the unique spines on both uncus and gnathos. Moreover, the new species differs from these latter species by the presence of the large dorsal spot, which is lacking in *P. longifurcata* and *P. spinosa*. Also see *Remark*.

*Barcodes*. We barcoded the male holotype collected in Cantarranas on 23 April 2023 and the female paratype collected in the same locality on 10 February 2024. The sequences have been deposited in the National Genomics Data Center (NGDC, China) (accession IDs: SAMC6087960 and SAMC6087961).

*Male* (Figure 10a,b,g). The forewing is 2.4 mm long; the wingspan is 5.4 mm (*n* = 1). The frontal tuft is short, snow white. The collar and the thorax are glossy snow white, but distally the thorax is marked with a distinct dark brown spot and the collar possesses an ochre shadow along the distal edge. The forewing is glossy snow white, with a large dorsal spot comprised of brown-black scales with strong blue-purple iridescence. The apical strigulae are triangularly curved, formed by black-brown or dark brown scales, with a large ochre shadow anteriorly. The apical dot is black, elongated, but weakly defined. The area between two strigulae is cream or white. The fringe varies from dark grey to ochre cream. The hindwing and its fringe are brown.

*Female* (Figure 10c–e). The forewing is 2.5 mm long; the wingspan is 5.6 mm (*n* = 1). The scaling of the female is similar to that of the male.

**Figure 17:**
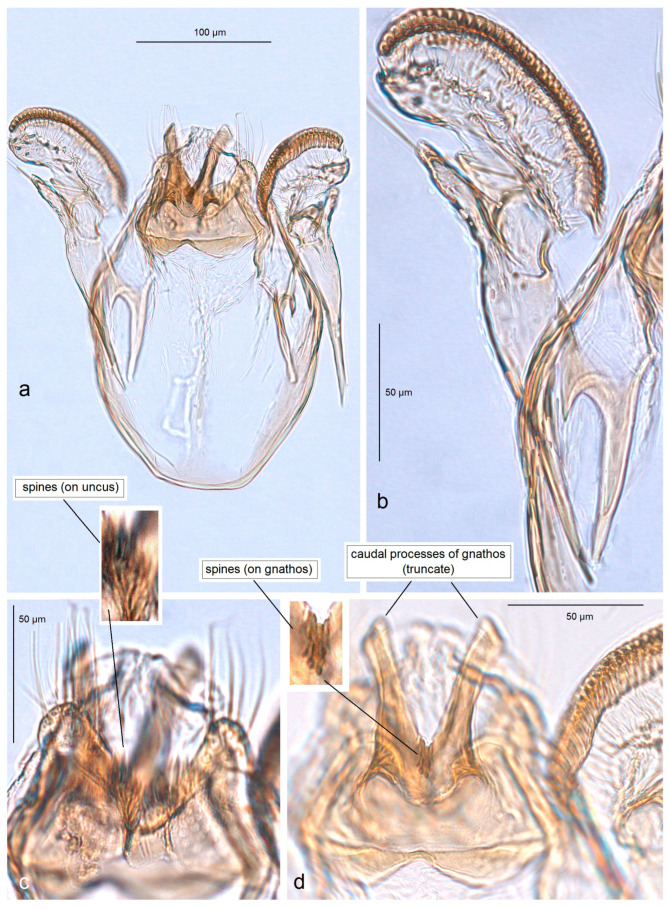
Male genitalia of *Pseudopostega bestiola* Stonis & Remeikis, sp. nov., holotype, genitalia slide no. RA1269 (MfN): (**a**) general view; (**b**) valva; (**c**) uncus; (**d**) gnathos.

*Male genitalia* (Figure 17). The capsule is 285 µm in length and 185 µm in width. The uncus possesses two large, distally rounded lateral lobes; the gap between the lobes is V-shaped and bears distinctive spines. The gnathos has smooth, laterally thickened lateral lobes and two distally truncated caudal processes; the area where the caudal processes furcate bears small unique spines. The vinculum is wide and truncated distally, without lateral lobes. The juxta is ill-defined.

**Figure 18:**
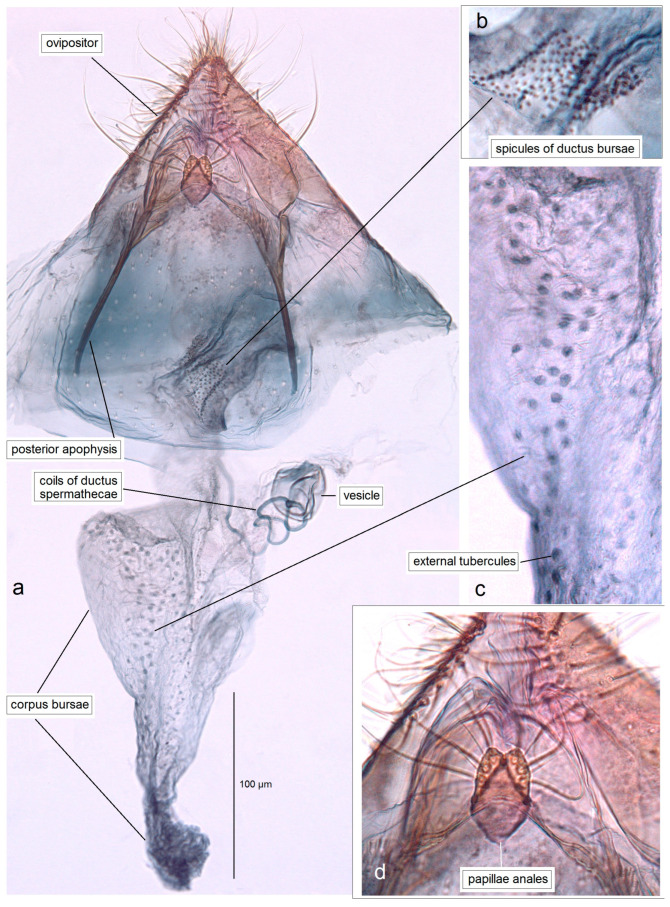
Female genitalia of *Pseudopostega bestiola* Stonis & Remeikis, sp. nov., paratype, genitalia slide no. RA1270 (MfN): (**a**) general view; (**b**,**c**) ductus bursae; (**d**) papillae anales.

*Female genitalia* (Figure 18). The total length is about 905 µm. The abdomen tapering to a distinctly triangularly shaped ovipositor. The posterior apophyses are slender. The anal papillae are bilobed, but the lobes are ill-defined, small, and closely set to each other. The vestibulum is relatively slender, membranous, and densely covered with spicules. The ductus bursae is wide and bears external tubercules arranged in a band. The ductus spermathecae is relatively short, with four large coils and a large rounded vesicle.

*Bionomics*. Adults are probably active from February to April (were detected in February and April), and fly to light. Otherwise, the species biology is unknown.

*Distribution*. The new species is known from only a single locality in Honduras: Cantarranas, Francisco Morazán Department, at elevations about 660–690 m.

*Material examined*. Holotype: ♂, Honduras, Francisco Morazán Department, Cantarranas (=San Juan de Flores), 660 m, 14°15′57″N, 87°01′31″W–14°16′17″N, 87°01′04″W, 18–23 April 2023, genitalia slide no. RA1269♂ (MfN). Paratype: 1 ♀, Cantarranas (=San Juan de Flores), 660–690 m, 14°16′17″N, 87°01′07″W–14°15′50″N, 87°01′35″W, 10–15 February 2024, genitalia slide no. RA1270♀ (MfN).

*Etymology*. The species name *bestiola* is a Latin noun meaning “little beast” (feminine, diminutive of *bestia*) and is treated as a noun in apposition. The name alludes to the striking appearance of the species, which bears a brown-black spot on the dorsum and spines on both the uncus and gnathos in the male genitalia—features that confer a somewhat roughened appearance compared to the majority of other *Pseudopostega* species.

*Remark*. The original grouping of *P. bidorsalis* [10] as separate from *P. longifurcata* and *P. spinosa* of the *P. brachybasis* group probably needs re-examination.

#### 3.2.4. *Pseudopostega merendoni* Stonis & Remeikis, sp. nov.

*ZooBank Registration*. https://zoobank.org/NomenclaturalActs/ffcafce1-b61b-4422-ac6a-218293203908 (accessed on 28 October 2025).

*Diagnosis*. The new species, *Pseudopostega merendoni* sp. nov., is assigned to the *P. lobata* species group (also see *Remark*). Externally, this new species can be confused with many other *Pseudopostega* species which possess a small dorsal spot, including those species discovered in Honduras. In the male genitalia, *P. merendoni* sp. nov. exhibits a unique gnathos with large, trapezoid lateral lobes and an extremely slender, rod-like caudal process.

*Barcode*. We barcoded the male holotype collected in San Pedro Sula (El Merendón) on 19 January 2023. The sequence has been deposited in the National Genomics Data Center (NGDC, China) (accession ID: SAMC6087962).

*Male* (Figure 11d,e). The forewing is 2.3 mm long; the wingspan is 5.2 mm (*n* = 1). The frontal tuft and the collar are snow white. The thorax is white but shaded with pale beige on most of the collar. The forewing is glossy snow white, with a small, slightly elongated dorsal spot of black-brown scales. The apical strigulae are ill-defined, brown-black; the apical dot is also ill-defined but black. The fringe is beige. The hindwing and its fringe are brown.

*Female*. The female of this species remains unknown.

**Figure 19:**
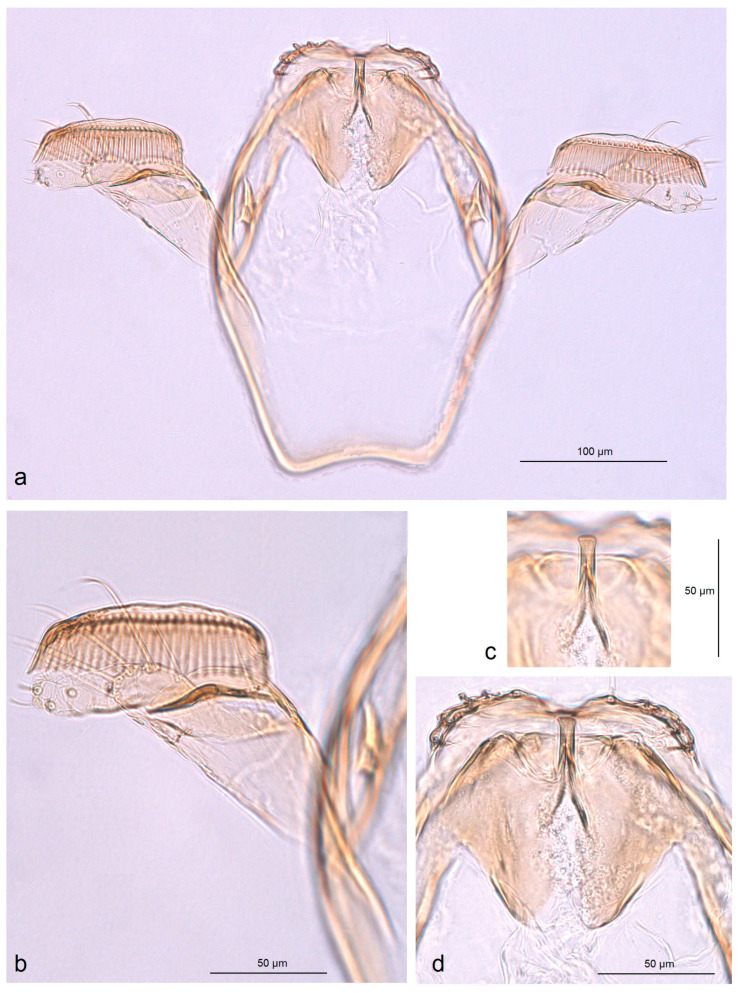
Male genitalia of *Pseudopostega merendoni* Stonis & Remeikis, sp. nov., holotype, genitalia slide no. RA1276 (MfN): (**a**) general view; (**b**) valva; (**c**,**d**) gnathos.

*Male genitalia* (Figure 19). The capsule is 300 µm in length and 210 µm in width. The uncus possesses two very wide and distally papillated lateral lobes; the gap between the lobes is small and shallow. The gnathos has smooth, very large, trapezoid lateral lobes, a shallow anterior excavation, and very slender, rod-like caudal processes arising from the middle of the plate-like gnathos; the length of the gnathos is 95 µm, while the width is 115 µm. The valva is 200 µm long; the cucullar lobe of the valva is 105 µm long. The vinculum is relatively wide but slightly tapering distally, with a very shallow anterior excavation and ill-defined lateral lobes. The juxta is absent.

*Bionomics*. Adults are active in January and fly towards light. Otherwise, the species biology is unknown.

*Distribution*. The new species is known from only a single locality in Honduras: El Merendón, San Pedro Sula, at an elevation of about 310 m.

*Material examined*. Holotype: ♂, Honduras, San Pedro Sula, El Merendon, 310 m, 15°31′08″N, 88°03′14″W, 19–20 January 2023, genitalia slide no. RA1276♂ (MfN).

*Etymology*. The species name is derived from the rather isolated mountain, El Merendón, on the eastern slope of which it was discovered.

*Remark*. The current placement of the new species in the *P. lobata* group was confirmed molecularly.

#### 3.2.5. *Pseudopostega mexicana* Remeikis & Stonis, 2009

*Barcodes.* We barcoded three specimens of presumably both sexes collected in February and March 2023 in Isla del Tigre and Isla Zacate Grande, the Pacific coast of Honduras. Their sequences have been deposited in the National Genomics Data Center (NGDC, China) (accession IDs: SAMC6087963 and SAMC6087964).

*Male* (Figure 11a–c). In the series from Honduras, the forewing ranges from 1.7 to 1.8 mm; the wingspan ranges from 3.9 to 4 mm (*n* = 4). For the diagnosis and detail species description, see the primary description [21]: pp. 282,283. The species is attributed to the *P. spatulata* species group [19,21].

*Female*. In the series from Honduras, the forewing is 2.1 mm in length; the wingspan is 4.7 mm (*n* = 1).

*Distribution*. Primary, the species was known only from Mexico, Oaxaca region (the Pacific coast of Mexico). It has now also been discovered in Honduras for the first time.

**Figure 20:**
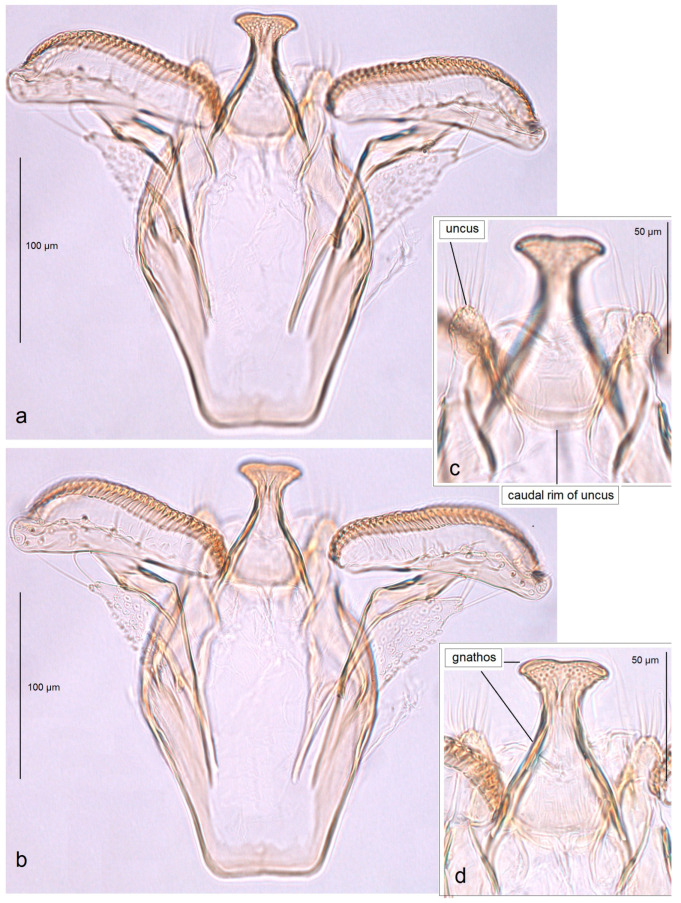
Male genitalia of *Pseudopostega mexicana* Remeikis & Stonis, recorded for the first time in Honduras, genitalia slide no. RA1271 (MfN): (**a**,**b**) general view; (**c**) uncus; (**d**) gnathos.

**Figure 21:**
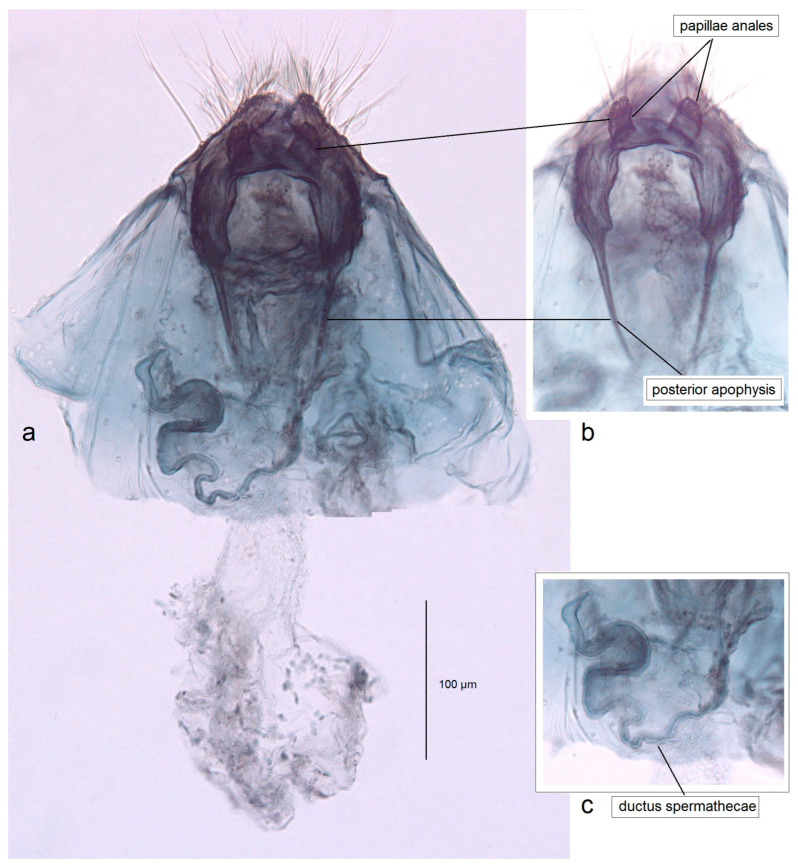
First documentation of female genitalia of *Pseudopostega mexicana* Remeikis & Stonis, discovered in Honduras for the first time, genitalia slide no. RA1283 (MfN): (**a**) general view; (**b**) apophyses and papillae anales; (**c**) ductus spermathecae.

*Material examined* (Figure 20 and Figure 21). 1 ♂, Honduras, Pacific coast, Isla del Tigre, Amapala, Playa Caracol, 20 m, 13°16′41″N, 87°39′29″W, 18 February 2023, leg. J.R. Stonis, genitalia slide no. RA1271♂ (MfN); 1 ♀, Pacific coast, Isla del Tigre, Amapala, Playa Grande, 40 m, 13°16′32″N, 87°39′37″W, 13–14 March 2023, leg. J.R. Stonis, genitalia slide no. RA1283♀ (MfN); 1 ♂, Pacific coast, Isla Zacate Grande, El Moray (Restaurante Terra Mar), 20 m, 13°21′28″N, 87°36′06″W, 15–16 February 2023, leg. J.R. Stonis (MfN); 2 ♀, Pacific coast, 1.5 km east by the Pan American Hwy (left side), 40 m, 13°25′59″N, 87°25′24″W, 6–14 February 2023, leg. J.R. Stonis (MfN).

*Remarks.* The females and DNA barcodes of this species were previously unknown; during the current study we discovered and documented the female of *P. mexicana* for the first time (Figure 21) and provide the species barcodes.

#### 3.2.6. *Pseudopostega adusta* (Walsingham, 1897)

*Barcodes.* We barcoded two specimens, one male and one female, collected on 8–10 April 2024 in Tela, Atlántida Department, Honduras. Their sequences have been deposited in the National Genomics Data Center (NGDC, China) (accession IDs: SAMC6087966 and SAMC6087967).

*Male* (Figure 11f). In the series from Honduras, the forewing ranges from 2.3 to 2.5 mm; the wingspan ranges from 5.3 to 5.7 mm (*n* = 8). For diagnosis and detailed species description, see the previous taxonomic revision by Davis and Stonis [10] as well as the primary description of *Opostega adusta* by Walsingham [40]: p. 140. The species is attributed to the *P. longipedicella* species group [10].

*Female*. In the series from Honduras, the forewing ranges from 2.2 to 2.9 mm; the wingspan ranges from 5.0 to 6.5 mm (*n* = 5).

*Distribution*. Prior to our studies, the species was known from the US Virgin Islands (St. Thomas), Cuba, Dominica, Belize [10], Ecuador: Napo Province, and Costa Rica: Pacific coast [21]. It has now also been discovered in Honduras for the first time.

**Figure 22:**
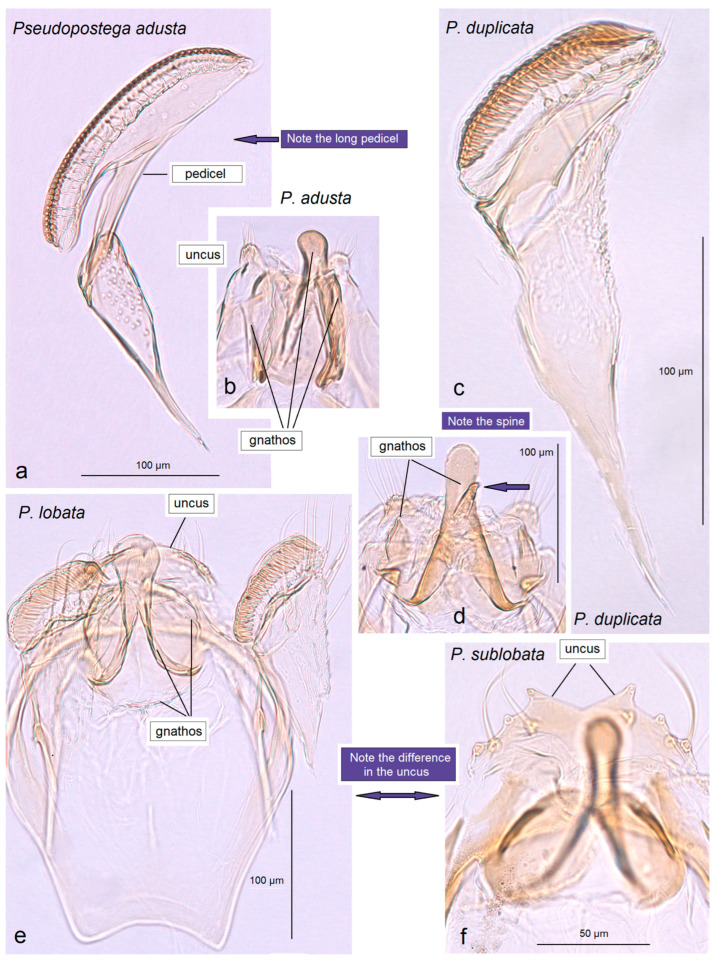
Comparison of the male genitalia of *Pseudopostega* Kozlov species discovered for the first time in Honduras: (**a**,**b**) *P. adusta*, RA1262; (**c**,**d**) *P. duplicata*, RA1273; (**e**) *P. lobata*, RA1265; (**f**) *P. sublobata*, RA1266 (MfN).

*Material examined* (Figure 22a,b). 1 ♂, Honduras, Atlántida Department, Tela, 10–30 m, 15°45′56″N, 87°27′06″W–15°46′09″N, 87°23′15″W, 2–19 April 2024, leg. J.R. Stonis, genitalia slide no. RA1262♂ (MfN). Additional 22 ♂ and 9 ♀ specimens collected at the same locality between February and April 2024 were also available for our study and are deposited at NRC due to a possible mix of the undissected series with *P. lobata* Davis & Stonis and *P. sublobata* Davis & Stonis.

#### 3.2.7. *Pseudopostega lobata* Davis & Stonis, 2007

*Barcodes.* We barcoded two specimens, one male and one female, collected on 2 and 13 April 2024 in Tela, Atlántida Department of Honduras. Their sequences have been deposited in the National Genomics Data Center (NGDC, China) (accession IDs: SAMC6087968 and SAMC6087969).

*Male* (Figure 11g,h). In the series from Honduras, the forewing ranges from 2 to 2.5 mm; the wingspan ranges from 4.6 to 5.6 mm (*n* = 2). For diagnosis and a detailed species description, see the previous taxonomic revision of Davis and Stonis [10], pp. 104–105. The species is attributed to the *P. lobata* species group [10].

*Distribution*. Prior to our studies, the species was known from Belize, Nicaragua, Costa Rica [10], Ecuador [21], and northwestern Argentina [10]. It has now also been discovered in Honduras for the first time.

*Material examined* (Figure 22e). 1 ♂, Honduras, San Pedro Sula, El Merendon, 310 m, 15°31′08″N, 88°03′14″W, 11 February 2023, genitalia slide no. RA1275♂ (MfN); 1 ♂, Atlántida Department, Tela, 10–30 m, 15°45′56″N, 87°27′06″W–15°46′09″N, 87°23′15″W, 2–19 April 2024, leg. J.R. Stonis, genitalia slide no. RA1265♂ (MfN). Additionally, a large series of specimens collected in Atlántida Department, Tela, during February–April 2024, representing a mix of undissected *P. lobata* Davis & Stonis, *P. sublobata* Davis & Stonis, and *P. adusta* (Walsingham), was available for our study (NRC).

#### 3.2.8. *Pseudopostega sublobata* Davis & Stonis, 2007

*Barcodes.* We barcoded two specimens, one male and one female, collected on 27 February and 2 April 2024 in Tela, Atlántida Department, Honduras. Their sequences have been deposited in the National Genomics Data Center (NGDC, China) (accession IDs: SAMC6087970 and SAMC6087971).

*Male* (Figure 11i,j). In the series from Honduras, the forewing ranges from 2.3 to 2.4 mm; the wingspan ranges from 5.2 to 5.4 mm (*n* = 2). For diagnosis and a detailed species description, see the previous taxonomic revision of Davis and Stonis [10]: pp. 107–108. The species is attributed to the *P. lobata* species group [10].

*Distribution*. Prior to our studies, the species was known from Costa Rica and Ecuador [10], as well as Peru [19]. It has now also been discovered in Honduras for the first time.

*Material examined* (Figure 22f). 1 ♂, 1 ♀, Honduras, San Pedro Sula, El Merendon, 310 m, 15°31′08″N, 88°03′14″W, 11 February 2023, genitalia slide no. RA1274♂ (MfN); 2 ♂, Atlántida Department, Tela, 10–30 m, 15°45′56″N, 87°27′06″W–15°46′35″N, 87°27′18″W, 27 February–19 March 2024, leg. J.R. Stonis, genitalia slide nos. RA1266♂ (no pinned specimen was preserved; it was taken for DNA extraction), RA1286♂ (MfN).

Additionally, a large series of specimens collected in Atlántida Department, Tela, during February–April 2024, representing a mix of undissected *P. sublobata* Davis & Stonis, *P. lobata* Davis & Stonis, and *P. adusta* (Walsingham), was available for our study (NRC).

#### 3.2.9. *Pseudopostega duplicata* Davis & Stonis, 2007

*Barcode.* We barcoded one male specimen collected on 18 April 2023 in Cantarranas, Francisco Morazán Department, Honduras. The sequence has been deposited in the National Genomics Data Center (NGDC, China) (accession ID: SAMC6087972).

*Male* (Figure 12a,b). In the series from Honduras, the forewing is 2.5 mm long; the wingspan is 5.7 mm (*n* = 1). For diagnosis and a detailed species description, see the previous taxonomic revision of Davis and Stonis [10]. The species is attributed to the *P. duplicata* species group [10].

*Distribution*. Prior to our studies, the species was known from Costa Rica and the British Virgin Islands [10]. It has now also been discovered in Honduras for the first time.

*Material examined* (Figure 22c,d). 1 ♂, Honduras, Francisco Morazán Department, Cantarranas (=San Juan de Flores), 660 m, 14°15′57″N, 87°01′31″W–14°16′17″N, 87°01′04″W, 18–23 April 2023, genitalia slide no. RA1273♂ (MfN).

#### 3.2.10. *Pseudopostega ocellata* Stonis & Remeikis, sp. nov.

*ZooBank Registration*. https://zoobank.org/NomenclaturalActs/d6f8ec3c-9e73-4b3c-b732-d3fb4f7efe3f (accessed on 28 October 2025).

*Diagnosis*. The new species, *Pseudopostega ocellata* sp. nov., is assigned to the *P. divaricata* species group. Externally, it is characterized by a distinct apical dot on the forewing, surrounded by a curved apical strigula. In the male genitalia, *P. ocellata* is distinguished from the most similar Cuban species, *P. turquinoensis* Davis & Stonis [10], by the rhomboid lateral lobes of the gnathos and a weak bifurcation of the caudal process.

*Barcode*. We barcoded one male paratype specimen collected on 27 February 2024 in Tela, Atlántida Department, Honduras. The sequence has been deposited in the National Genomics Data Center (NGDC, China) (accession ID: SAMC6087980).

*Male* (Figure 12c,d). The forewing length ranges from 2.6 to 2.7 mm; the wingspan ranges from 5.7 to 6.2 mm (*n* = 2). The frontal tuft is short and snow white. The collar and the thorax are glossy snow white. The forewing is glossy snow white, lacking dorsal or costal spots and without fascia. The first costal strigula is wider and dark brown; the second strigula is slender and black; the apical strigula is brown and broadly curves around the apical dot, which is large and comprises black scales. The fringe is beige cream. The hindwing and its fringe appear grey at certain angles but glossy pale brownish cream when viewed from other angles.

*Female*. The female of this species remains unknown.

**Figure 23:**
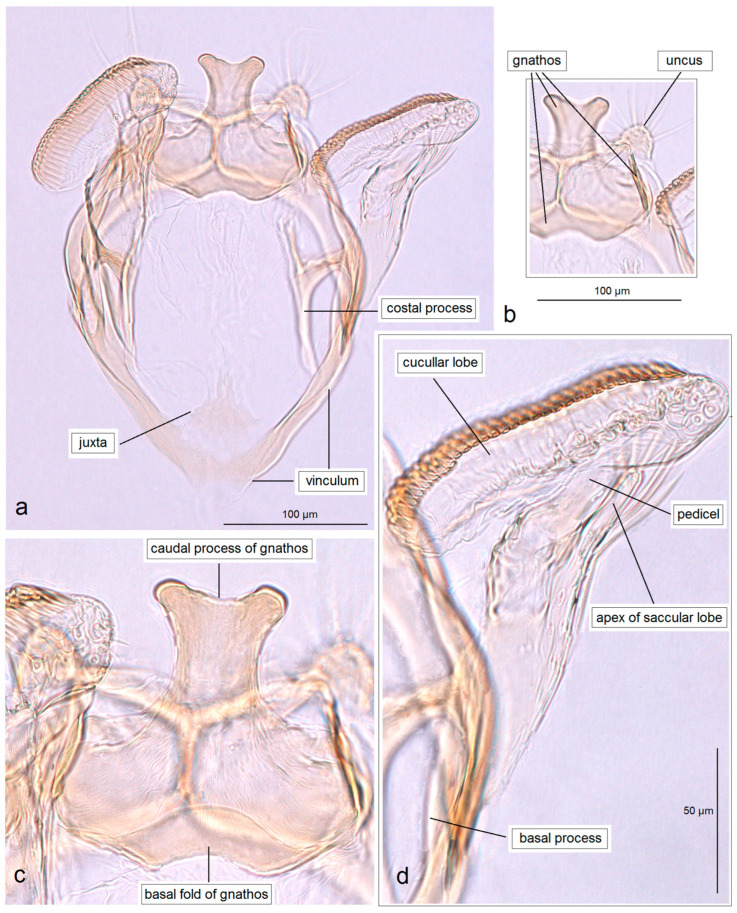
Male genitalia of *Pseudopostega ocellata* Stonis & Remeikis, sp. nov., holotype, genitalia slide no. RA1264 (MfN): (**a**) general view; (**b**) uncus and gnathos; (**c**) gnathos; (**d**) valva.

*Male genitalia* (Figure 23). The capsule ranges from 250 to 305 µm in length and from 190 to 220 µm width. The uncus possesses two well-separated, distally rounded lateral lobes. The gnathos has thickened rhomboid lateral lobes and a wide, distally bifid caudal process. The valva measures about 175–195 µm in length; the cucullar lobe ranges from 125 to 130 µm long. The vinculum is distinctly tapered distally, pointed or truncated, without lateral lobes. The juxta is ill-defined, short but wide.

*Bionomics*. Adults are active from February to March and are attracted to light. Otherwise, the species’ biology is unknown.

*Distribution*. The new species is known only from a single locality in Honduras: Tela, Atlántida Department, along the Caribbean coast, at elevations from 10 to 30 m.

*Material examined*. Holotype: ♂, Honduras, Atlántida Department, Tela, 10–20 m, 15°45′56″N, 87°27′06″W–15°46′35″N, 87°27′18″W, 27 February–19 March 2024, leg. J.R. Stonis, genitalia slide no. RA1264♂ (MfN). Paratype: 1 ♂, the same label data as the holotype, genitalia slide no. RA1263♂ (MfN).

*Etymology*. The species name *ocellata* is a Latin feminine adjective meaning “marked with little eyes” or “eye-spotted”. It refers to the eye-like pattern at the apex of the forewing, where the combination of pale apical strigulae and a relatively large black apical dot gives the appearance of a single ocellus.

#### 3.2.11. *Pseudopostega pumila* (Walsingham, 1914)

*Barcodes.* We barcoded seven specimens of both sexes collected in February–April 2023 and 2024 in Tela, Atlántida Department, Honduras. Their sequences have been deposited in the National Genomics Data Center (NGDC, China) (accession IDs: SAMC6087973–SAMC6087979).

*Male* (Figure 12e,f). In the Honduran series, the forewing ranges from 2.8 to 2.9 mm; the wingspan ranges from 5.5 to 6.2 mm (*n* = 3). For the species description, see the previous taxonomic revision of Davis and Stonis [10] as well as the original description of *Opostega pumila* by Walsingham [41]: p. 350. The species is attributed to the *P. brachybasis* group [10].

*Distribution*. Prior to our studies, the species was known only from Mexico (Tabasco) [10], but it has now also been discovered in Honduras.

**Figure 24:**
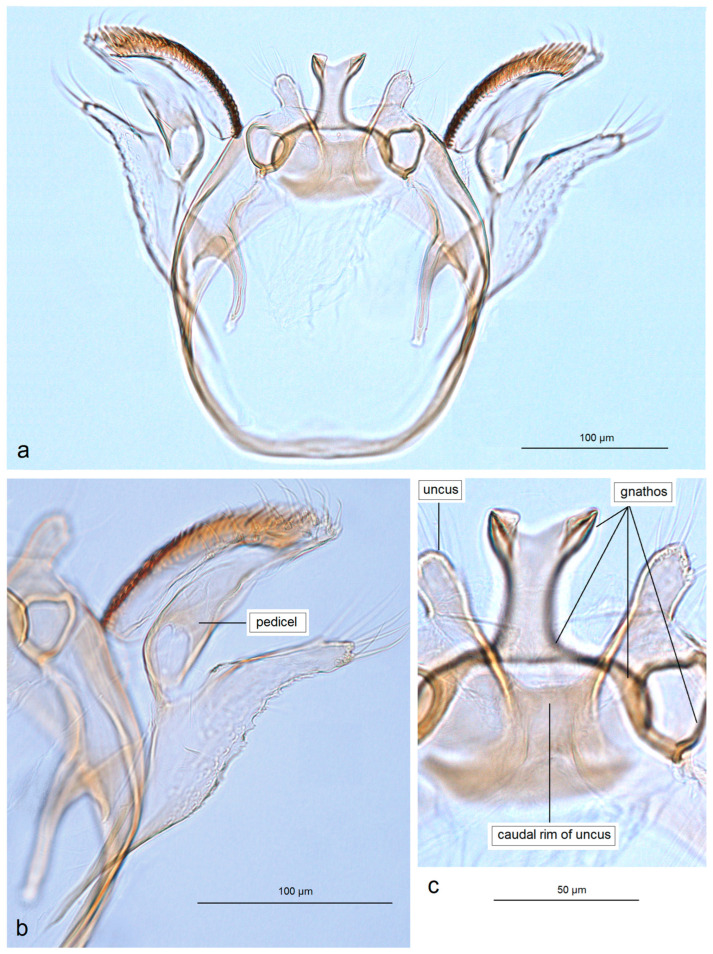
Male genitalia of *Pseudopostega pumila* (Walsingham), recorded for the first time in Honduras, slide no. RA1267 (MfN): (**a**) general view; (**b**) valva; (**c**) uncus and gnathos.

*Material examined* (Figure 24). 3 ♂, Honduras, Atlantída Department, Tela, 10–30 m, 15°45′56″N, 87°27′06″W, 27 February–19 March 2024, leg. J.R. Stonis, genitalia slide nos. RA1267♂ (no pinned specimen was preserved; it was taken for DNA extraction), RA1272♂, RA1285♂ (MfN); 3 ♂, the same locality, 2–19 April 2024, leg. J.R. Stonis (MfN); 1 ♀, Atlantída Department, Tela, 20 m, 15°46′0″N, 87°27′14″W, 9–10 April 2023, leg. J.R. Stonis (MfN); 1 ♀, Atlantída Department, 7.5 km south east of La Ceiba, Villas Pico Bonito, 100 m, 15°43′40″N, 86°44′29″W, 21 January 2023, leg. J.R. Stonis (MfN).

### 3.3. Molecular Considerations

In this study, we successfully obtained mtDNA CO1-5′ barcodes for all 11 Opostegidae species recorded in Honduras, resulting in thirty three 657 bp-long sequences in total. Among them, seven sequences were generated for each of *Pseudopostega cristagalli* sp. nov. and *P. pumila* (Walsingham), five for *P. geometra* sp. nov., three for *P. mexicana* Remeikis & Stonis, and two for each of *P. adusta* (Walsingham), *P. bestiola* sp. nov., *P. lobata* Davis & Stonis, and *P. sublobata* Davis & Stonis. Single sequences were obtained for *P. merendoni* sp. nov., *P. ocellata* sp. nov., and *P. duplicata* Davis & Stonis. Importantly, the dataset includes the first DNA barcode for *P. mexicana*, a species described from Mexico in 2009 [21]. All sequences are considered reliable (“verified”), as DNA was extracted either from type series specimens of new species or from reliably identified material confirmed through detailed genitalia examinations.

**Figure 25:**
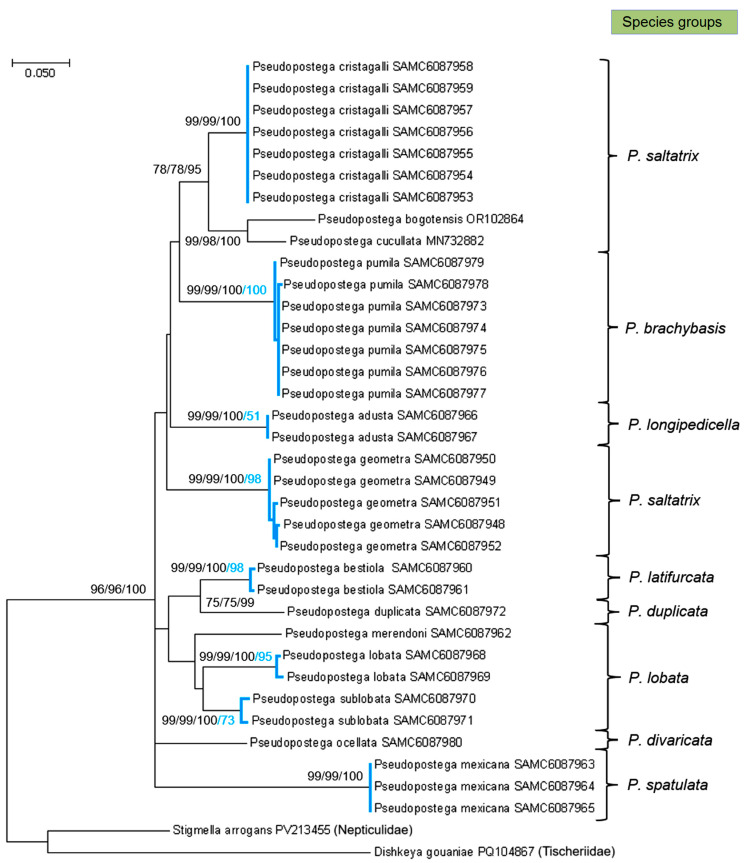
The topology of the analysed *Pseudopostega* Kozlov species based on the 657 bp-long mtDNA CO1 sequences. Numbers represent the bootstrap values obtained for Neighbor-Joining (10,000 replicates)/Maximum Likelihood (10,000 replicates)/Bayesian inference (10,000,000 generations)/bPTP (blue) in %. Values below 50 are not shown. *Dishkeya gouaniae* (Stonis & Diškus) and *Stigmella arrogans* Stonis & Diškus were included as an outgroup.

Interestingly, one male specimen that had been identified as *P. ocellata* in the field and preserved in ethanol was later shown, through sequencing, to belong to *P. pumila*. This case highlights the risk of misidentification when relying solely on external adult morphology—particularly under field conditions—and underscores the necessity of examining genitalia for accurate species determination prior to DNA barcoding. Fortunately, in the laboratory we were able to extract DNA from the dry hind leg of the paratype of *P. ocellata*, and thus we were able to include the barcode of this new species in the publication. All sequences obtained in this study are presented in Figure 25, together with two previously generated *Pseudopostega* Kozlov sequences from Colombia (*P. cucullata* Stonis & Vargas and *P. bogotensis* Vargas) [19] and two outgroup sequences: one from the sister family Nepticulidae (*Stigmella arrogans* Stonis & Diškus) [23] and another from the more distantly related Tischeriidae (*Dishkeya gouaniae* Stonis & Diškus) [32]. Phylogenetic trees were constructed using three different approaches—Neighbor-Joining (NJ), Maximum Likelihood (ML), and Bayesian inference. As DNA barcodes are intended to delimitate species [42], but not resolve deeper phylogenetic relationships, the basal dichotomies in our tree remain unsupported. Though species delimitation was justified by the updated Poisson tree processes algorithm (bPTP), which estimates speciation in terms of the number of substitutions [37] and expresses species differences in terms of Bayesian support values. Accordingly, the tree in Figure 25 should not be viewed as a reconstruction of species-level phylogeny, but rather as an illustration of the distinctness of the newly described and newly analyzed species.

Molecular analyses demonstrated that all Opostegidae species discovered in Honduras are well differentiated from one another (Figure 25). The calculated pairwise distances showed that the interspecific distance between studied species varied from 7.28 ± 1.23% (between *P. lobata* H14R and *P. sublobata* RA1266) to 16.79 ± 2.25% (between *P. duplicata* and *P. mexicana*). The largest intraspecific variability was determined in *P. sublobata*, where it reached 0.93 ± 0.38% between HLOB2 and RA1266 sequences. These results are consistent with the findings of the study that established a 3% CO1 threshold between different lepidopteran species [43]. Some of the analysed species whose more than one sequence have been obtained did not exhibit intraspecific variability (*P. adusta*, *P. cristagalli* sp. nov., *P. mexicana*), though this might change as more specimens are studied. For example, between the *P. geometra* sp. nov. sequences presented in this paper, two (RA1277 and RA1281) are identical, while the other three are each characterized by unique mitotype. Along with subtle morphological differences in male genital structures (vinculum and uncus) and adult forewing apical strigulae, molecular characteristics distinguish *P. geometra* sp. nov. as a distinct and well-defined (bPTP—95%), but somewhat variable, species.

The type series of *P. bestiola* sp. nov. was limited to a single male (holotype) and a single female (paratype), both collected in the same locality (Cantarranas) and indistinguishable in external morphology. Assigning a previously unknown female, captured at light, to a new species is typically considered risky and generally avoided without molecular support. In this case, however, the molecular data provided strong confirmation (bPTP—99%) that the sequences of the female and male, collected in a different year and at a different time, belong to the same species (Figure 25).

Opostegidae, including species of the genus *Pseudopostega*, are commonly arranged into species groups [9,10,19,21]. For comparative purposes, sequences of additional Opostegidae species from certain *Pseudopostega* groups were retrieved from BOLD [33]. It helped to assign the newly described *P. merendoni* sp. nov. species to the *P. lobata* group (Figure 25). This finding is significant, because before molecular evidence was available, the unique morphological features of *P. merendoni* sp. nov. made it difficult to determine whether the species was more closely related to the *P. spatulata* group or to the *P. lobata* group.

At present, including the 33 new sequences generated in this study, and two previously obtained sequences from Colombia [19], a total of 114 Neotropical Opostegidae mtDNA CO1-5′ sequences, representing 19 species of *Pseudopostega* and 5 *Neopostega* Davis & Stonis species, are available [31,33]. Considering the importance of molecular barcodes as a tool in taxonomic lepidopterans studies [44,45,46,47,48], this number of published sequences must be regarded as highly insufficient, as it represents only 23.3% of the described Neotropical *Pseudopostega* and *Neopostega* species.

**Figure 26:**
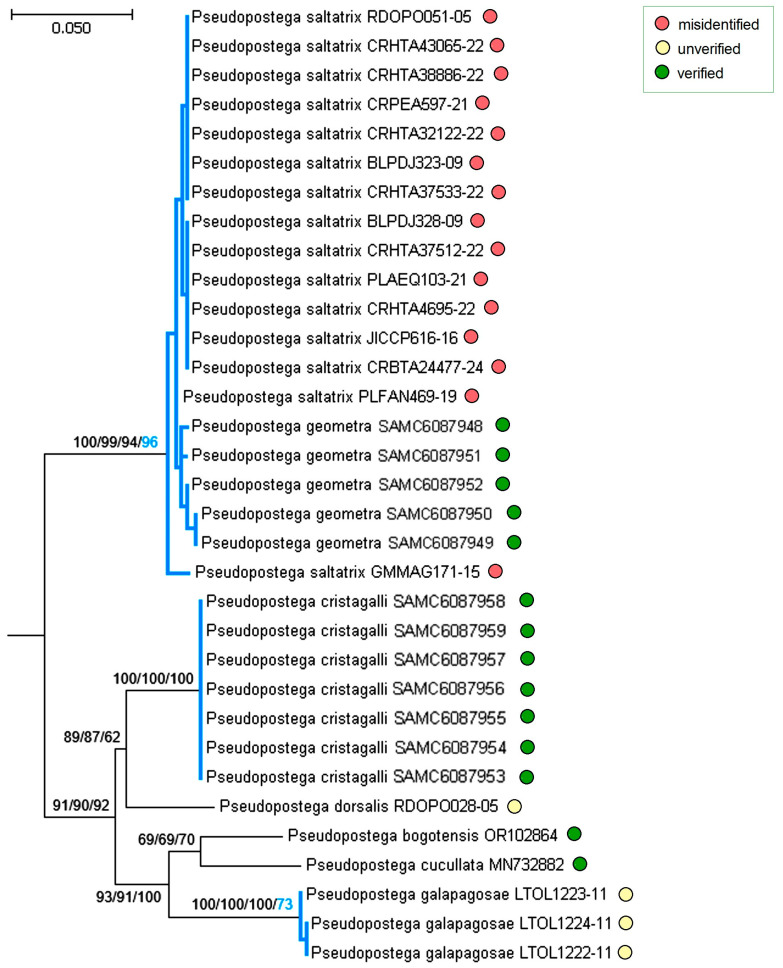
The topology of the *Pseudopostega saltatrix* species group based on the 657 bp long mtDNA CO1 sequences. Numbers represent the bootstrap values obtained for Neighbor-Joining (10,000 replicates)/Maximum Likelihood (10,000 replicates)/Bayesian inference (10,000,000 generations)/bPTP (blue) in %. Values below 50 are not shown. *Dishkeya gouaniae* (Stonis & Diškus) (Tischeriidae) and *Stigmella arrogans* Stonis & Diškus (Nepticulidae) were included as an outgroup.

**Figure 27:**
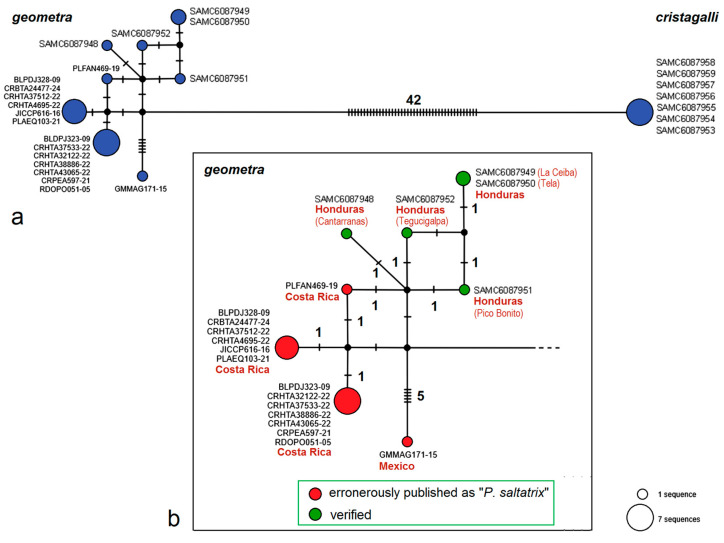
The mitotype network of 525 bp long mtDNA CO1 sequences of *Pseudopostega* Kozlov species, constructed using the TCS Network algorithm. Circle size is proportional to the frequency of each mitotype. Smaller, unnamed circles represent predicted (hypothetical) mitotypes. Dashes on the lines connecting mitotypes indicate hypothesized mutational steps: (**a**) *P. geometra* sp. nov. and related *P. cristagalli* sp. nov.; (**b**) *P. geometra* sp. nov., enlarged.

Moreover, some published sequences raised doubts. Analysis of the *P. saltatrix* species group (Figure 26 and Figure 27) revealed that 14 sequences from Costa Rica and one from Mexico, deposited in the BOLD platform under the name “*P. saltatrix*”, are surprisingly similar to those of *P. geometra* sp. nov., described here as new. These 15 sequences, labeled as “*P. saltatrix*”, along with our paratype sequences of *P. geometra* sp. nov. form the distinct monophyletic clade, which is well supported not only by genetic distance-based methods (NJ—100%, ML—99%, Bayesian inference—94%), but also by the applied species delimitation test (bPTP—96%). The pairwise evolutionary distances of these sequences ranged from 0% within mitotypes to 2.21 ± 0.91% (=nine substitutions, between, e.g., RA1280 and GMMAG171-15), while the variability between *P. geometra* sp. nov.–“*P. saltatrix*” and other related species from the same species group fluctuated from 8.39 ± 1.44% (with *P. dorsalis*) to 12.24 ± 1.75% (with *P. galapagosae*), which shows the significant barcoding gap between them. A mitotype network depicting the distribution of moderately distant *P. cristagalli* sp. nov. and *P. geometra* sp. nov.–“*P. saltatrix*” (average distance: 9.50 ± 1.40%, 44–47 substitutions) highlights the genetic uniqueness and internal similarity of the latter group. Although no molecular data are available for true *P. saltatrix* (Walsingham), the first part of our study provided a clear diagnosis of this species based on genitalia and external morphology, demonstrating that it is distinctly different from *P. geometra* sp. nov. Thus, the 15 BOLD sequences attributed to “*P. saltatrix*” represent a typical case of misidentification.

**Figure 28:**
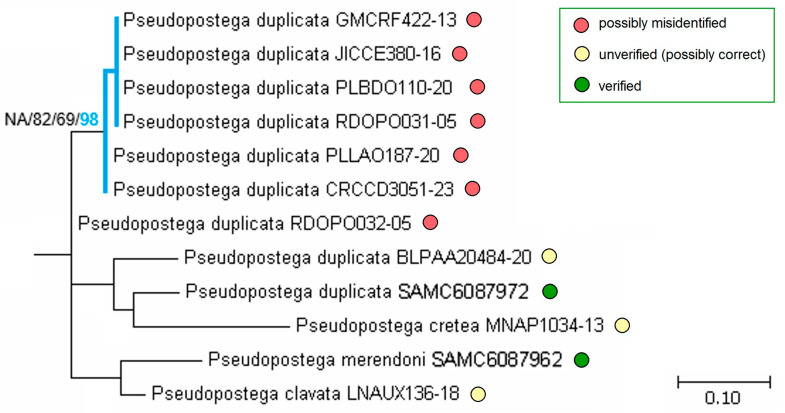
The topology of the *Pseudopostega duplicata* Davis & Stonis and other selected species based on the 657 bp long mtDNA CO1 sequences. Numbers represent the bootstrap values obtained for Neighbor-Joining (10,000 replicates)/Maximum Likelihood (10,000 replicates)/Bayesian inference (10,000,000 generations)/bPTP (blue) in %. Values below 50 are not shown. *Dishkeya gouaniae* (Stonis & Diškus) (Tischeriidae) and *Stigmella arrogans* Stonis & Diškus (Nepticulidae) were included as an outgroup.

While analyzing sequences of the *P. duplicata* species group (Figure 28), and comparing them with sequences from other groups, we found that all eight sequences published prior to our study under the name “*P. duplicata*” did not form a supported clade with our reliably identified *P. duplicata* Davis & Stonis (verified through male genitalia). On the contrary, part of them (six sequences) grouped into a well resolved monophyletic clade (ML—82%, Bayesian inference—69%), which was treated as a separate species (bPTP—98%). This leads to the assumption that all sequences labeled as “*P. duplicata*” are most likely erroneous and some of them represent other, not *P. duplicata*, species’ material.

**Figure 29:**
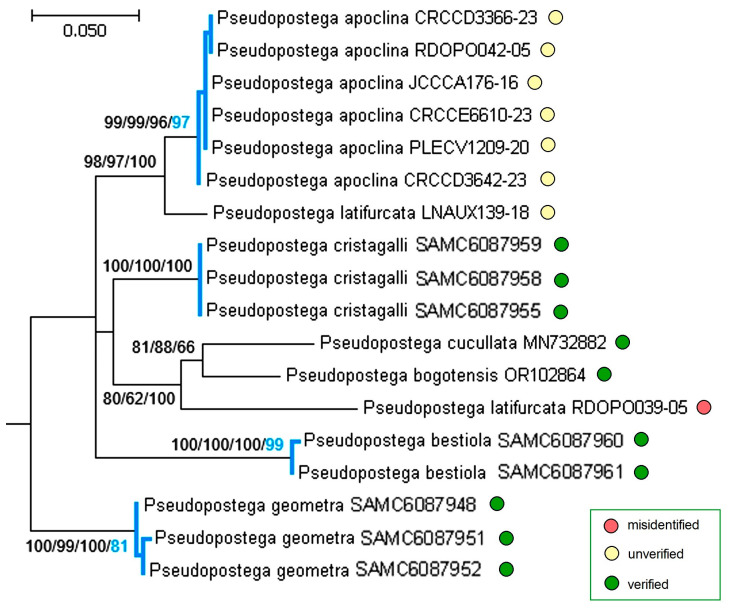
The topology of the *Pseudopostega latifurcata* species group and other selected species based on the 657 bp long mtDNA CO1 sequences. Numbers represent the bootstrap values obtained for Neighbor-Joining (10,000 replicates)/Maximum Likelihood (10,000 replicates)/Bayesian inference (10,000,000 generations)/bPTP (blue) in %. Values below 50 are not shown. *Dishkeya gouaniae* (Stonis & Diškus) (Tischeriidae) and *Stigmella arrogans* Stonis & Diškus (Nepticulidae) were included as an outgroup.

In the tree, that included all molecularly available species of the *P. latifurcata* group (Figure 29), together with comparative taxa from other groups, the two previously published sequences of *P. latifurcata* Davis & Stonis from the British Virgin Islands differed markedly (the evolutionary distance between them was 11.48 ± 2.32%), which definitely means that at least one of them was presumably the result of misidentification. It is possible—although not confirmed by our study—that sequence LNAUX139-18 was identified correctly, as it reliably clustered as a sister taxon to *P. apoclina*, a species within the *P. latifurcata* group (NJ—98%, ML—97%, Bayesian inference—100%). By contrast, the sequence RDOPO039-05 formed a quite well-defined clade with morphologically distant species of the *P. saltatrix* group (NJ—80%, ML—62%, Bayesian inference—100%).

**Figure 30:**
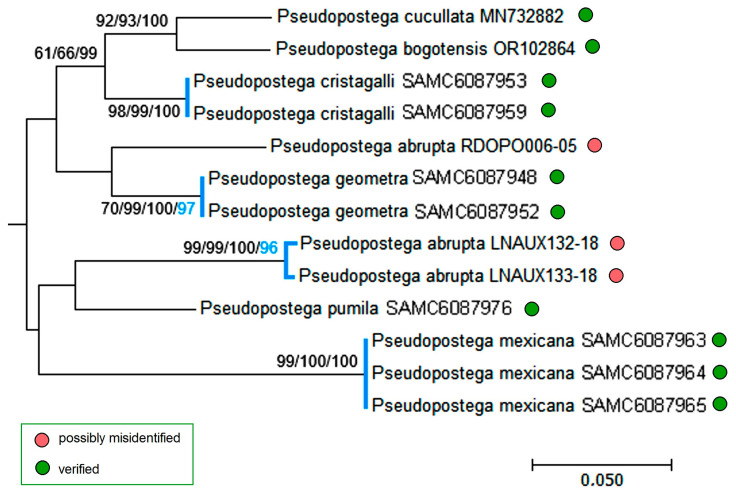
The topology of the *Pseudopostega lateriplicata* species group and selected species based on the 657 bp long mtDNA CO1 sequences. Numbers represent the bootstrap values obtained for Neighbor-Joining (10,000 replicates)/Maximum Likelihood (10,000 replicates)/Bayesian inference (10,000,000 generations)/bPTP (blue) in %. Values below 50 are not shown. *Dishkeya gouaniae* (Stonis & Diškus) (Tischeriidae) and *Stigmella arrogans* Stonis & Diškus (Nepticulidae) were included as an outgroup.

A similar case was observed in the *P. lateriplicata* species group (Figure 30): three previously published sequences of *P. abrupta* occupied radically different, though not reliable, positions in the tree, suggesting that at least two of them—LNAUX132-18 and LNAUX133-18—forming the distinct monophyletic clade (NJ—99%, ML—99%, Bayesian inference—100%), supported as a separate species (bPTP—96%), are questionable, and one of them (RDOPO006-05) is presumably erroneous.

**Figure 31:**
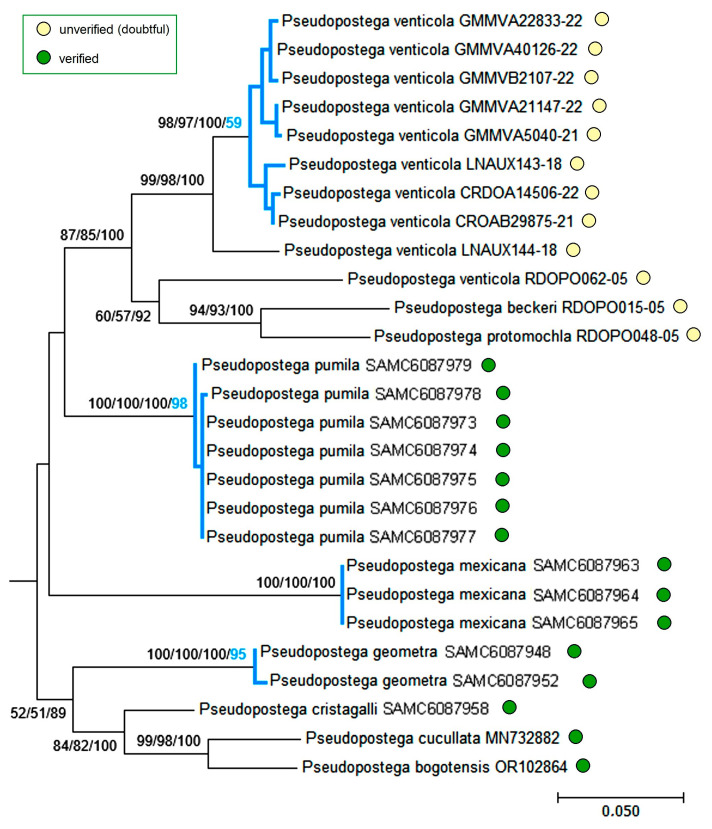
The topology of the *Pseudopostega brachybasis* species group and selected species based on the 657 bp long mtDNA CO1 sequences. Numbers represent the bootstrap values obtained for Neighbor-Joining (10,000 replicates)/Maximum Likelihood (10,000 replicates)/Bayesian inference (10,000,000 generations)/bPTP (blue) in %. Values below 50 are not shown.

In the tree presented in Figure 31, the *P. brachybasis* species group along with the *P. spatulata* group is separated from the *P. saltatrix* group, which forms the lowest clade. The two latter clades, composed entirely of verified sequences, raise no concerns. By contrast, the upper clade, representing the *P. brachybasis* group, consists of mixed sequences. Within this clade, verified *P. pumila* sequences formed a well-resolved monophyletic group (NJ, ML, Bayesian inference—100% each), confirmed as a distinct species (bPTP—98%). Meanwhile, the topology of the rest sequences labeled as “*P. beckeri*” Davis & Stonis, “*P. protomochla*” (Meyrick), “*P. venticola*”, though being well supported, raises doubts about their correct identification. Eight of ten sequences published as “*P. venticola*” merged into a distinct clade, and were treated as a separate species (bPTP—59%), while the sequence LNAUX144-18 was not included, and the sequence RDOPO062-05 clustered as a sister group to the sequences under the names “*P. beckeri*” and “*P. protomochla*”.

**Figure 32:**
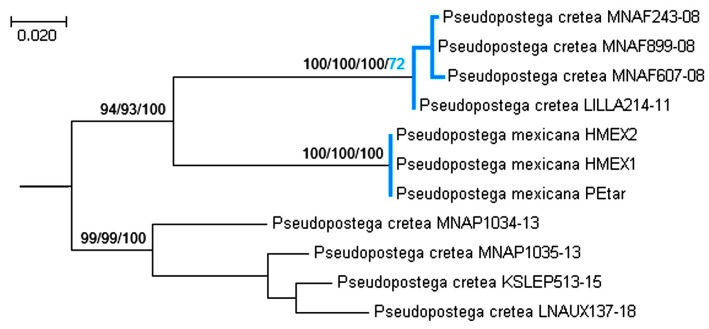
The topology of all currently available *Pseudopostega cretea* (Meyrick) and *P. mexicana* Remeikis & Stonis sequences. Numbers represent the bootstrap values obtained for Neighbor-Joining (10,000 replicates)/Maximum Likelihood (10,000 replicates)/Bayesian inference (10,000,000 generations)/bPTP (blue) in %.

Although *Pseudopostega cretea* (Meyrick) is a boreal species and was not included in our checklist of Neotropical taxa, records under the name “*P. cretea*” also exist from Texas and Florida, USA. The molecular sequences published in the BOLD platform under this name formed a paraphyletic group (Figure 32), showing exceptional variability in our analysis: the evolutionary distances fluctuated from 0.15 ± 0.15% (between MNAF243-08 and MNAF899-08) to 23.66 ± 3.27% (between LILLA214-11 and LNAUX137-18). Therefore, it would be incorrect to assume that all sequences published as “*P. cretea*” belong to a single species.

### 3.4. Updating the Checklist of Central America and the Caribbean Opostegidae with an Expanded Neotropical Coverage

The current checklist of Opostegidae includes 63 species from Central America and the Caribbean, incorporating recent discoveries, as well as species from the remainder of the Neotropical region (i.e., South America). Altogether, the checklist covers 103 species, some of which have overlapping distributions in Central and South America.

The Neotropical region is treated here in the broad sense, i.e., without adopting the recent separation of the non-tropical Ando-Patagonian region (for current biogeographical concepts see Morrone [49,50] and Stonis et al. [51,52]). If considered separately, the Ando-Patagonian region would contain only a single species, *Notiopostega atrata* Davis, which belongs to a highly distinctive Valdivian endemic genus.

In the current list, three species from Florida, USA, with a clear Neotropical affiliation are also included: *Pseudopostega kempella* (Eyer), *P. parakempella* Davis & Stonis, and *P. floridensis* Davis & Stonis. These species are excluded from the count of Nearctic fauna in the subsequent *Discussion* section. Similarly, *P. acidata* (Meyrick, 1915), described from Ecuador [53] but recorded along the Gulf of Mexico coast in Texas (USA) near the Mexican border [10], is here treated as exclusively Neotropical and not included in the Nearctic count. In contrast, *P. venticola* Walsingham, a species widespread in the Neotropics but also recorded from Florida and Texas [10], is included in both the Neotropical and Nearctic fauna counts.

*Pseudopostega cretea* (Meyrick, 1920) [54], mentioned in our section on *Molecular Considerations*, was not included in the current Neotropical checklist because its distribution is primarily boreal, ranging from Canada (Ontario, British Columbia) to the United States (Louisiana, Connecticut, Michigan, Mississippi, Nebraska, Pennsylvania, Washington, Maine, North Carolina, Wisconsin, and Texas) [10]. Its occurrence in Florida [33] remains to be verified; in any case, the presence of a boreal species in Florida does not “automatically” qualify it as Neotropical.

The terminology and concept of species groups follow our previous publication [19]. Distribution data for the numerous species described by Davis and Stonis (the first author of the present article) are based on material examined during the revision of the American Opostegidae [10] and are not individually cited in the checklist below. DNA barcodes of the species were obtained from the BOLD database [33].

 **Genus *Notiopostega* Davis, 1989** ***Notiopostega atrata*** Davis, 1989  *Distribution*. The new species is known from Chile (Valdivia) [10]. **Genus *Neopostega* Davis & Stonis, 2007** ***Neopostega longispina*** Davis & Stonis, 2007  *Distribution.* The species is known from the lowland Amazonian rainforest of southern Venezuela. ***Neopostega falcata*** Davis & Stonis, 2007  *Distribution.* The species is known from northeastern Costa Rica (Heredia). ***Neopostega asymmetra*** Davis & Stonis, 2007  *Distribution.* The species is known from the Atlantic coastal forest of southern Brazil. ***Neopostega petila*** Davis & Stonis, 2007  *Distribution.* The species is known from northeastern Costa Rica (Heredia). ***Neopostega distola*** Davis & Stonis, 2007  *Distribution.* The species is known from northern Costa Rica (Alad) and southwestern Brazil (Mato Grosso). ***Neopostega nigrita*** Heppner & Davis, 2009  *Distribution.* The species is known from Guatemala [11]. ***Neopostega dondavisi*** Stonis & Remeikis, 2020  *Distribution.* The species is known from a single locality: La Merced, Junín Region, central Peru, at an elevation of about 900 m (selva central/selva alta) [19]. **Genus *Pseudopostega* Kozlov, 1985** The *rotunda* group ***Pseudopostega rotunda*** Davis & Stonis, 2007  *Distribution.* The species is known from Costa Rica (Heredia and Guanacaste), and the Amazonian rainforest of Ecuador (Napo Province). ***Pseudopostega ovatula*** Davis & Stonis, 2007  *Distribution.* The species is known from the Amazonian lowland and premontane rainforest of Ecuador (Napo Province). ***Pseudopostega attenuata*** Davis & Stonis, 2007  *Distribution.* The species is known from northeastern Costa Rica (Heredia, Cartago, and Guanacaste), northwestern Brazil (Ceara), and southwestern Ecuador (Bucay). ***Pseudopostega latisaccula*** Davis & Stonis, 2007  *Distribution.* The species is known from Dominica (NW Pont Casse) and Puerto Rico (Carite). The *latifurcata* group ***Pseudopostega latifurcata*** Davis & Stonis, 2007  *Distribution.* The species is known from US Virgin Islands (St. Thomas), Puerto Rico (Cayey, Carite), British Virgin Islands (Tortola Island), and Dominica (Cabrit Swamp). ***Pseudopostega apoclina*** Davis & Stonis, 2007 (=*P. latifurcata apoclina* Davis & Stonis, 2007).  *Distribution.* The species is known from Costa Rica (Guanacaste, Cartago). ***Pseudopostega bidorsalis*** Davis & Stonis, 2007  *Distribution.* The species is known from northern Costa Rica (Heredia, Cartago). ***Pseudopostega bestiola*** Stonis & Remeikis, sp. nov. (described herein).  *Distribution.* The species is known from a single locality in Honduras: Cantarranas, Francisco Morazán Department, at elevations of about 660–690 m. The *lateriplicata* group ***Pseudopostega lateriplicata*** Davis & Stonis, 2007  *Distribution.* The species is known from northeastern Costa Rica (Heredia). ***Pseudopostega floridensis*** Davis & Stonis, 2007  *Distribution.* The species is known from southern Florida, USA. ***Pseodopostega robusta*** Remeikis & Stonis, 2009  *Distribution.* The species is known from the Pacific coast of Costa Rica [21]. ***Pseudopostega abrupta*** (Walsingham, 1897)  *Distribution.* The species is known from the US Virgin Islands (St. Thomas) and British Virgin Islands (Guana Island). ***Pseudopostega ferruginea*** Davis & Stonis, 2007  *Distribution.* The species is known from the US Virgin Islands (St. Thomas) and Puerto Rico. ***Pseudopostega uncinata*** Davis & Stonis, 2007  *Distribution.* The species is known from north-central Venezuela (Guarico). ***Pseudopostega serrata*** Davis & Stonis, 2007  *Distribution.* The species is known from Costa Rica (Heredia, Cartago, Limón, and Puntarenas), southern Panama, and the Amazonian rainforest of Ecuador (Napo Province). ***Pseudopostega monstruosa*** Davis & Stonis, 2007  *Distribution.* The species is known from the Amazonian premontane rainforest of Ecuador (Napo Province). The *triangularis* group ***Pseudopostega triangularis*** Davis & Stonis, 2007  *Distribution.* The species is known from northern Argentina (Salta). ***Pseudopostega conicula*** Davis & Stonis, 2007  *Distribution.* The species is known from northwestern Costa Rica (Guanacaste). ***Pseudopostega sacculata*** (Meyrick, 1915)  *Distribution.* The species is known from Ecuador (Chimborazo: Huigra; Guayas: Bucay) [53]; recently, it was discovered in the Central Amazonian forest of Peru (La Merced) [19]. ***Pseudopostega fumida*** Davis & Stonis, 2007  *Distribution.* The species is known from the tropical humid forest of Belize (Cayo District). ***Pseudopostega breviapicula*** Davis & Stonis, 2007  *Distribution.* The species is known from Panama (La Chorrera), Brazil (Pará, Minas Gerais), and northern Argentina (Jujuy). ***Pseudopostega mignonae*** Davis & Stonis, 2007  *Distribution.* The species is known from Jamaica (St Catherine Parish) and Cuba (Pinar del Rio). ***Pseudopostega acuminata*** Davis & Stonis, 2007  *Distribution.* The species is known from northern Venezuela (Mérida) and northern Argentina (Tucumán). ***Pseudopostega trinidadensis*** (Busck, 1910)  *Distribution.* The species is known from Trinidad and Tobago (Trinidad). ***Pseudopostega plicatella*** Davis & Stonis, 2007  *Distribution.* The species is known from northeastern Brazil (Pará) and the Amazonian rainforest of Ecuador (Napo Province). ***Pseudopostega subtila*** Davis & Stonis, 2007  *Distribution.* The species is known from southeastern Brazil (Minas Gerais). ***Pseudopostega kempella*** (Eyer, 1967)  *Distribution.* The species is known from Monroe County of southern Florida, USA [55]. ***Pseudopostega paraplicatella*** Davis & Stonis, 2007  *Distribution.* The species is known from the Amazonian rainforest of Ecuador (Napo Province). ***Pseudopostega gracilis*** Davis & Stonis, 2007  *Distribution.* The species is know from the rainforest of French Guiana. ***Pseudopostega tanygnatha*** Davis & Stonis, 2007  *Distribution.* The species is known from northwestern Costa Rica (Guanacaste). The *spatulata* group ***Pseudopostega spatulata*** Davis & Stonis, 2007  *Distribution.* The species is known from northeastern Costa Rica (Heredia). ***Pseudopostega truncata*** Davis & Stonis, 2007  *Distribution.* The species is known from central Brazil (Distrito Federal: Maranhao). ***Pseudopostega mexicana*** Remeikis & Stonis, 2009  *Distribution.* The species is known from the Pacific coast of Mexico (Oaxaca region) [21]. In the present article, we provide new distributional data from the Pacific coast of Honduras. ***Pseudopostega apotoma*** Davis & Stonis, 2007  *Distribution.* The species is known from Brazil (Minas Gerais and Pará); recently it has also been recorded from the Amazonian rainforest of Peru (Satipo) [19]. ***Pseudopostega pexa*** (Meyrick, 1920)  *Distribution.* The species is known from northeastern Brazil (Pará) [54]. ***Pseudopostega diskusi*** Davis & Stonis, 2007  *Distribution.* The species is known from the tropical humid forest of Belize (Cayo District). ***Pseudopostega microacris*** Davis & Stonis, 2007  *Distribution.* The species is known from northeastern Costa Rica (Heredia and Cartago). ***Pseudopostega tucumanae*** Davis & Stonis, 2007  *Distribution.* The species is known from northern Argentina (Tucumán). The *saltatrix* group ***Pseudopostega colognatha*** Davis & Stonis, 2007  *Distribution.* The species is known from Puerto Rico (Patillas and Carite). ***Pseudopostega obtusa*** Davis & Stonis, 2007  *Distribution.* The species is known from Ecuador (Carchí). ***Pseudopostega pontifex*** (Meyrick, 1915)  *Distribution.* The species is known from western Colombia (Cali) [54]. ***Pseudopostega cucullata*** Stonis & Vargas, 2020  *Distribution.* The species is known from a single locality in Colombia: Valle del Cauca, on the border of the National Park de los Farallones de Cali, at an elevation of about 1700 m (Desarrollo Biodiverso) [19]. ***Pseudopostega bogotensis*** Vargas, 2020  *Distribution.* The species is known from green urban areas of Bogotá, Colombia, at an elevation of about 2600 m [19]. ***Pseudopostega galapagosae*** Davis & Stonis, 2007  *Distribution.* The species is known from the Galápagos Islands, Ecuador. ***Pseudopostega saltatrix*** (Walsingham, 1897)  *Distribution.* The species is known from the US Virgin Islands (St. Thomas) [40]. All other previously reported records from the Caribbean and Central and South America [10] are most likely incorrect. ***Pseudopostega dorsalis*** Davis & Stonis, 2007 (=*P. dorsalis dorsalis* Davis & Stonis, 2007)  *Distribution.* The species is known from Costa Rica (San José and Cartago). ***Pseudopostega fasciata*** Davis & Stonis, 2007 (=*P. dorsalis fasciata* Davis & Stonis, 2007)  *Distribution.* The species is known from Costa Rica (Alajuela, Cartago, Heredia, and Guanacaste). ***Pseudopostega parakempella*** Davis & Stonis, 2007  *Distribution.* The species is known from Florida, USA [10], and the Pacific coast of Mexico (Oaxaca region: Puerto Angel) [21]. ***Pseudopostega latiplana*** Remeikis & Stonis, 2009  *Distribution.* The species is known from the Pacific coast of Mexico (Oaxaca region: Puerto Angel) [21]. ***Pseudopostega jamaicensis*** Stonis & Remeikis, sp. nov. (described herein)  *Distribution.* The species is known from Jamaica. ***Pseudopostega geometra*** Stonis & Remeikis, sp. nov. (described herein)  *Distribution.* The species is known from various localities of Honduras; the published DNA sequences also indicated that this species is in Costa Rica. ***Pseudopostega cristagalli*** Stonis & Remeikis, sp. nov. (described herein)  *Distribution.* The species is known from the tropical humid forest of northern Honduras (Tela). The *longipedicella* group ***Pseudopostega longipedicella*** Davis & Stonis, 2007  *Distribution.* The species is known from Costa Rica (Puntarenas) and Panama (Canal Zone). ***Pseudopostega adusta*** (Walsingham, 1897)  *Distribution.* The species is known from the US Virgin Islands (St. Thomas) [40], Cuba (Central Baragua), Dominica (Cabrit Swamp), Belize (Cayo District) [10]. It has also been recorded from the Pacific coast of Costa Rica [21] and from Ecuador (Napo Province) [21]. In the present article, we provide new distributional data from Honduras. The *lobata* group ***Pseudopostega lobata*** Davis & Stonis, 2007  *Distribution.* The species is known from Costa Rica (Heredia and Guanacaste), Belize (Cayo District), Nicaragua (Estelí) [10], Ecuador (Napo Province) [21], and Argentina (Jujuy and Salta) [10]. In the present article, we provide new distributional data from Honduras. ***Pseudopostega clavata*** Davis & Stonis, 2007  *Distribution.* The species is known from southeastern Puerto Rico (Cayey). ***Pseudopostega sublobata*** Davis & Stonis, 2007  *Distribution.* The species is known from Costa Rica (Heredia), Ecuador (Bucay) [10], and Peru (Satipo: selva central) [19]. In the current article, we provide new distribution data from Honduras. ***Pseudopostega merendoni*** Stonis & Remeikis, sp. nov. (described herein).  *Distribution.* The species is known from northwestern Honduras (El Merendon). The *duplicata* group ***Pseudopostega duplicata*** Davis & Stonis, 2007  *Distribution.* The species is known from Costa Rica (Heredia, Cartago, Guanacaste, and Limón) and the British Virgin Islands (Tortola Island). In the present article, we provide new distributional data from Honduras. ***Pseudopostega didyma*** Davis & Stonis, 2007  *Distribution.* The species is known from the western slopes of the Andes (Bucay) in Ecuador [10]; recently, it was also discovered in the Amazonian rainforest of Ecuador (Napo Province) [21]. ***Pseudopostega acidata*** (Meyrick, 1915)  *Distribution.* The species is known from southern Ecuador (Chimborazo: Huigra) [53], as well as the USA (Texas) [10] but the latter record needs to be re-examined. ***Pseudopostega tenuifurcata*** Davis & Stonis, 2007  *Distribution.* The species is known from northeastern Costa Rica (Heredia). ***Pseudopostega sectila*** Davis & Stonis, 2007 *Distribution*. The species is known from the British Virgin Islands (Tortola Island) and Puerto Rico (Maricao). The *microlepta* group ***Pseudopostega microlepta*** (Meyrick, 1915)  *Distribution.* The species is known from Guyana (Mazaruni-Potaro) and Ecuador (Duran) [53]. The *divaricata* group ***Pseudopostega curtarama*** Davis & Stonis, 2007  *Distribution.* The species is known from southern Brazil (Minas Gerais and Goias). ***Pseudopostega crassifurcata*** Davis & Stonis, 2007  *Distribution.* The species is known from southeastern Cuba (Sierra Maestra). ***Pseudopostega turquinoensis*** Davis & Stonis, 2007  *Distribution.* The species is known from southeastern Cuba (Santiago). ***Pseudopostega concava*** Davis & Stonis, 2007  *Distribution.* The species is known from northeastern Mexico (Tamaulipas). ***Pseudopostega acrodicra*** Davis & Stonis, 2007  *Distribution.* The species is known from southern Brazil (Minas Gerais). ***Pseudopostega caulifurcata*** Davis & Stonis, 2007  *Distribution.* The species is known from southwestern Brazil (Mato Grosso). ***Pseudopostega contigua*** Davis & Stonis, 2007  *Distribution.* The species is known from Venezuela (Teritorio Federal de Amazonas). ***Pseudopostega divaricata*** Davis & Stonis, 2007  *Distribution.* The species is known from northern Argentina (Jujuy). ***Pseudopostega denticulata*** Davis & Stonis, 2007  *Distribution.* The species is known from the Amazonian rainforest of Ecuador (Napo Province). ***Pseudopostega brevifurcata*** Davis & Stonis, 2007  *Distribution.* The species is known from northern Costa Rica (Heredia and Guanacaste). ***Pseudopostega brevivalva*** Davis & Stonis, 2007  *Distribution.* The species is known from Costa Rica (Heredia). ***Pseudopostega resimafurcata*** Davis & Stonis, 2007  *Distribution.* The species is known from southeastern Brazil (Minas Gerais). ***Pseudopostega ocellata*** Stonis & Remeikis, sp. nov. (described herein)  *Distribution.* The species is known from the Caribbean coast of northwestern Honduras. The *brachybasis* group ***Pseudopostega ecuadoriana*** Davis & Stonis, 2007  *Distribution.* The species is known from the Amazonian rainforest of Ecuador (Napo Province). ***Pseudopostega beckeri*** Davis & Stonis, 2007  *Distribution.* The species is known from southern and central Brazil (Goias, Minas Gerais, and Paraná). ***Pseudopostega protomochla*** (Meyrick, 1935)  *Distribution.* The species is known from northern Argentina (Córdoba, Catamarca, Salta, and Tucumán) and southern Brazil (Minas Gerais) [10,56]. ***Pseudopostega venticola*** (Walsingham, 1897) *Distribution*. The species is known from Puerto Rico, Dominica, Grenada (Balthazar), Mexico (Campeche), Costa Rica (Guanacaste), Panama (Cabima), Venezuela (Guarico), Brazil (Mato Grosso and Pará), and Ecuador (Napo Province) [10,40], as well as from Florida and Texas, USA [10]. Recently, the species was also recorded from the central Amazonian rainforest of Peru (Satipo) [19]. ***Pseudopostega longifurcata*** Davis & Stonis, 2007  *Distribution.* The species is known from Jamaica (St. Catherine Parish). ***Pseudopostega spinosa*** Stonis and Diškus, 2020  *Distribution.* The species is known from two areas on opposite sides of the Ecuadorian Andes: the western Andean foothills at an elevation of about 700 m, and the premontane Amazonian rainforest at an elevation of about 500 m [19]. ***Pseudopostega monosperma*** (Meyrick, 1931)  *Distribution.* The species is known from eastern Brazil (Bahia) [57]. ***Pseudopostega suffuscula*** Davis & Stonis, 2007  *Distribution.* The species is known from northern Argentina (Salta and Tucumán). ***Pseudopostega bicornuta*** Davis & Stonis, 2007  *Distribution.* The species is known from southern Mexico (Chiapas). ***Pseudopostega brachybasis*** Davis & Stonis, 2007  *Distribution.* The species is known from northeastern Mexico (Tamaulipas). ***Pseudopostega constricta*** Davis & Stonis, 2007  *Distribution.* The species is known from southern Mexico (Chiapas). ***Pseudopostega bifida*** Stonis & Remeikis, 2020  *Distribution.* The species is known from the central selva of Peru (Departamento de Junín: Satipo), at an elevation of about 750 m [19]. ***Pseudopostega latiapicula*** Davis & Stonis, 2007  *Distribution.* The species is known from Costa Rica (Heredia) and Brazil (Paraná). ***Pseudopostega pumila*** (Walsingham, 1914)  *Distribution.* The species is known from Mexico (Tabasco) [41]. In the present article, we provide new distributional data from Honduras. Ungrouped species ***Pseudopostega congruens*** (Walsingham, 1914)  *Distribution.* The species is known from southwestern Mexico (Guerrero) [41]. ***Pseudopostega elachista*** (Walsingham, 1914)  *Distribution.* The species is known from southwestern Mexico (Guerrero) [41]. ***Pseudopostega paromias*** (Meyrick, 1915)  *Distribution.* The species is known from western Peru (Lima) [53]. ***Pseudopostega perdigna*** (Walsingham, 1914)  *Distribution.* The species is known from southwestern Mexico (Guerrero) [41].

## 4. Discussion

*Pseudopostega saltatrix* is historically the most widely recognized and “famous” species among Neotropical Opostegidae. It has long been erroneously considered a species with an exceptionally broad distribution range across the Neotropics, with numerous specimens from Belize and Costa Rica to Paraguay identified and even illustrated under this name [10]. However, the type material of *P. saltatrix* deposited at the Natural History Museum, London (NHMUK), had never been properly examined or documented. As a result, both morphological misinterpretations and numerous barcode misidentifications accumulated in public databases such as BOLD and GenBank.

Our recent study of the *P. saltatrix* holotype and paratype at NHMUK has revealed that all previously recorded specimens attributed to this species were misidentified, as the true *P. saltatrix* differs distinctly in morphology from those documented earlier. Importantly, the examination of type material also led to the discovery and description of an additional, previously unrecognized species.

A detailed re-examination of historical material deposited in the collection of the Natural History Museum, London, together with the first documentation of the Caribbean *Pseudopostega saltatrix* (Walsingham), has revealed that this species is not conspecific with the taxon (or taxa) previously published or identified under the same name from Central and South America. This clarification resolves a long-standing source of confusion in the taxonomy of Neotropical Opostegidae, ensuring that true *P. saltatrix* can be accurately distinguished from superficially similar but unrelated taxa. The present documentation of the type series of *P. saltatrix* not only provides a reliable basis for future identifications but also contributes to the stability of nomenclature and the accuracy of biodiversity records across the region.

**Figure 33:**
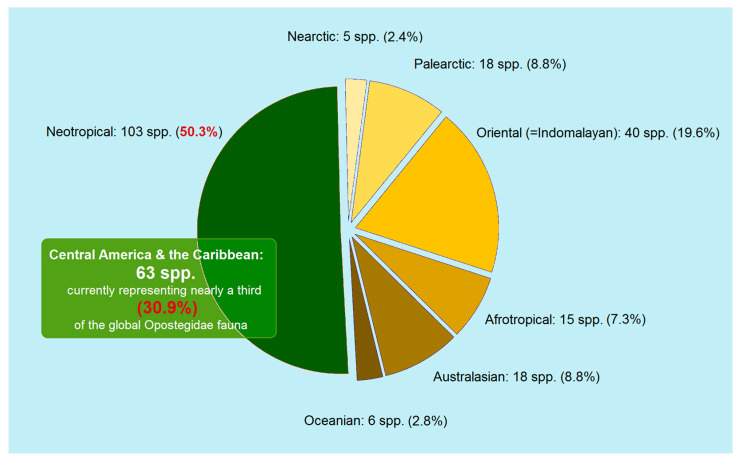
Current count of described and named Opostegidae species for each biogeographical region worldwide. Note that the count for the Oriental region excludes six species documented by Puplesis et al. [9] but not yet formally named. The following three species from Florida, USA, and one species from the Gulf of Mexico coast near the border with Mexico are included in the Neotropical count but excluded from the Nearctic count: *Pseudopostega kempella* (Eyer), *P. parakempella* Davis & Stonis, *P. floridensis* Davis & Stonis, and *P. acidata* (Meyrick). In contrast, *P. venticola* Walsingham, a species widespread in the Neotropics but also recorded from Florida and Texas, USA [10], is included in the counts of both the Neotropical and Nearctic faunas. *P. cretea* (Meyrick, 1920) [54], however, was excluded from the Neotropical count because its distribution is predominantly boreal North American.

With the discovery of six new species—*Pseudopostega jamaicensis* sp. nov., *P. geometra* sp. nov., *P. cristagalli* sp. nov., *P. merendoni* sp. nov., *P. bestiola* sp. nov., and *P. ocellata* sp. nov.—the number of Opostegidae species recorded from Central America and the Caribbean has risen to 63 (Figure 33). This total currently represents nearly a third (30.9%) of the global Opostegidae fauna, underscoring the region’s exceptional importance as a center of diversity for the family. The assemblage not only reveals an extraordinary radiation of *Pseudopostega* Kozlov species but also encompasses the majority of the known species of the Neotropical endemic genus *Neopostega* Davis & Stonis.

Central America and the Caribbean are subregions of the Neotropical biogeographical realm [49], which, when compared with another major realm, the Oriental, shows a striking difference (Figure 33): the Neotropics (103 species) significantly surpass the Oriental region (40 species, including one species recently described [58]), despite both being largely tropical. Remarkably, the Central America and Caribbean fauna alone already exceeds the entire Oriental fauna, suggesting particularly strong diversification of Opostegidae in the Neotropics. Such concentrations of species richness and certain endemicity highlight Central America and the Caribbean as a critical focal point for the study of Opostegidae evolution, biogeography, and conservation, and suggest that the true diversity of the group in this region may be even greater than currently recognized.

Possible drivers of this pattern may include the region’s complex geological and climatic history, as well as the biotic interchange between North and South America following the formation of the Isthmus of Panama. The hypothesis that Central America and the Caribbean could represent a center of origin for at least *Pseudopostega* Kozlov and *Neopostega* Davis & Stonis genera also cannot be easily dismissed. In contrast to the Neotropics, boreal regions hold drastically fewer species: the Palearctic biogeographical realm (18 species) and the Nearctic realm (5 species) show much lower diversity, consistent with the general macroecological gradient of increasing richness toward the tropics. The disparity is so pronounced that Central America and the Caribbean alone are 3.5 times richer in species than the entire Palearctic fauna. It should be noted that North America—and especially Europe—are likely close to their inventory limits due to long-term, intensive sampling, whereas Central America, the Caribbean (as demonstrated by the results of this study), and large parts of South America remain under-sampled, making it probable that the true Neotropical diversity of Opostegidae is substantially higher than the 103 species recorded to date.

**Figure 34:**
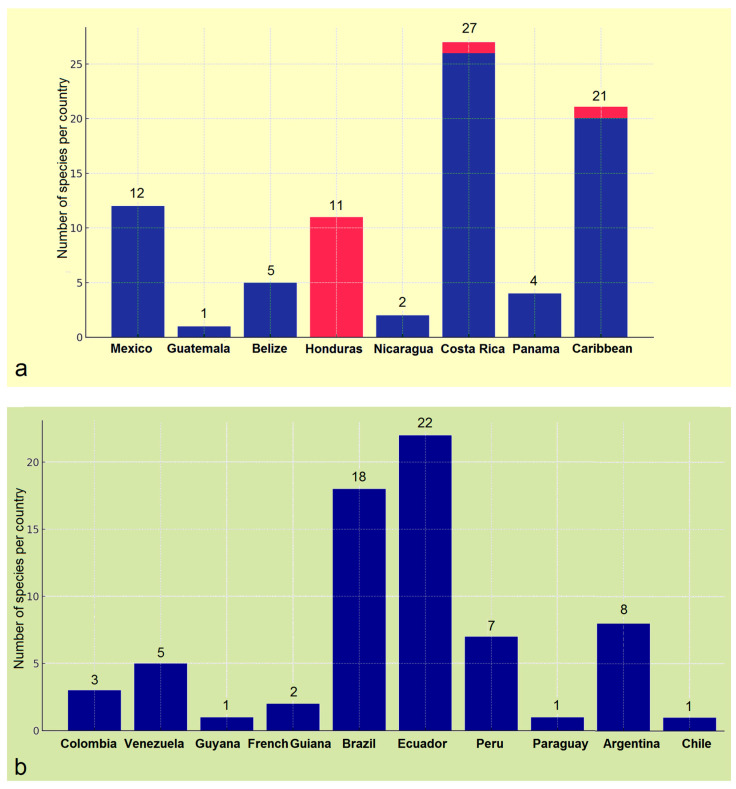
Currently described diversity of Opostegidae in the Neotropical region by country (countries not shown in the figure have no recorded Opostegidae to date): (**a**) Central America and the Caribbean, based on the Global Opostegidae Database 1997–2025 compiled by Arūnas Diškus (Vilnius, BRG) and the first two authors of this study. Some species occur in more than one country; therefore, totals in the graph exceed the 63 species currently known from the region. Caribbean islands are considered collectively. New discoveries provided by the present study are shown in red; (**b**) South America (updated from Stonis et al. [19]). Again, some species occur in more than one country; therefore, totals in the graph exceed the total number of species recorded from the continent.

Currently, largely due to differences in sampling effort, species richness is unevenly documented among the countries of Central America and the Caribbean (Figure 34a). Costa Rica, long recognized for its exceptional biodiversity and relatively intensive sampling, leads with 27 recorded species. Mexico (12 species) and Honduras (11 species) also support notable diversity, while Belize (5 species), Panama (4 species), Nicaragua (2 species), and Guatemala (1 species) contribute smaller numbers—likely reflecting a combination of genuine distributional patterns and uneven surveying intensities. It should be noted that countries not included in Figure 34 currently have no recorded Opostegidae at all. The Caribbean islands, considered here collectively, harbor 21 species, underscoring their significant role in the subregion’s overall diversity. These figures almost certainly underestimate the true national faunas, as many areas remain poorly explored. Figure 34b presents species records per country in South America, with Ecuador leading at 22 recorded species, followed by Brazil with 18 species. This figure allows for a comparison of the sampling effort between Central America and the Caribbean and South America, offering a broader perspective on the discovered Opostegidae diversity across the Neotropical region.

However, under-sampling is not the only problem. Although the family of white pygmy moths can be recognized relatively easily, species-level identification is a very different matter. In many cases, species are extremely similar and presumably young. Without prior experience in identifying Opostegidae, species determination can be highly unreliable. Moreover, diagnostic structures critical for identification can only be examined with a high-power microscope, while a standard field stereomicroscope is insufficient. Ideally, reliable species identification could be supported by molecular barcoding. Unfortunately, the availability of Opostegidae barcodes remains critically low. In the present study, we estimated that only about a third (32%) of Neotropical species have any DNA barcode data. Furthermore, among 114 sequences from the Neotropics deposited in BOLD and GenBank, only about 40% could be considered genuinely reliable; the others are unverified. Moreover, during the current study, we found a considerable proportion (about 27% of currently available sequences) to be obviously erroneous (some, but not all, were shown in Figure 26, Figure 27, Figure 28, Figure 29, Figure 30 and Figure 31). Erroneous barcodes may result from species misidentification, cross-contamination, sequencing errors, or inaccurate data submission (see [33,42,43]). In Opostegidae, however, we suggest that species misidentification could be the principal cause. This is an alarming situation since incorrectly identified barcodes inevitably generate further downstream problems.

It is our hope that this publication will encourage further investigations into Neotropical Opostegidae and foster a greater appreciation of the remarkable diversity within this often-overlooked group of microlepidoptera. Species inventory is not only a complex and responsible task, but also an engaging and rewarding process that deepens our understanding of biodiversity. During the final stage of preparation of this manuscript, a paper on the Opostegidae of China was published [58]. It described two new *Opostega* Zeller species: *O. apicidissecta* Sun & Wang and *O. magnirotunda* Sun & Wang. The latter species, from Hainan in the Oriental region, is included in our species counts, while some uncertainty remains regarding *O. apicidissecta*, which was described on the basis of a singleton from Xinjiang, due to its close resemblance to our specimens of *O. spatulella* Herrich-Schäffer from Central Asia.

## 5. Conclusions

(1) With the discovery of six new species—*Pseudopostega jamaicensis* sp. nov., *P. geometra* sp. nov., *P. cristagalli* sp. nov., *P. bestiola* sp. nov., *P. merendoni* sp. nov., and *P. ocellata* sp. nov.—the number of Opostegidae species recorded from Central America and the Caribbean has reached 63.

(2) A detailed re-examination of historical material deposited in the collection of the Natural History Museum, London, together with the first documentation of the Caribbean *Pseudopostega saltatrix* (Walsingham), has revealed that this species is not conspecific with the taxon (or taxa) previously published or identified under the same name from Central and South America. The present documentation of the type series of *P. saltatrix* should help prevent future misidentifications.

(3) Despite being a relatively small subregion, Central America and the Caribbean represents a global hotspot for Opostegidae. It currently contains nearly one-third (30.9%) of the world’s Opostegidae fauna and demonstrates not only a remarkable diversity of *Pseudopostega* Kozlov species, but also includes the majority of species belonging to the Neotropical endemic genus *Neopostega* Davis & Stonis.

(4) The Neotropical fauna currently comprises 103 species, including 63 from Central America and the Caribbean, 51 from South America, and a subset (10.7%) occurring in both regions—a proportion likely to rise with future research.

(5) In general, the Neotropical realm exhibits markedly higher Opostegidae diversity than other biogeographical realms, such as the tropical Oriental (40 spp.) and the boreal Palearctic (18 spp.), underscoring its importance as a center of diversification for white pygmy moths.

(6) Species richness is unevenly documented among the Neotropical countries, reflecting both genuine distributional patterns and differences in sampling effort, with Costa Rica, Mexico, and previously *tabula rasa* Honduras showing the highest recorded diversity, while several areas remain under-sampled.

(7) Our molecular analysis indicated that among 114 mtDNA CO1-5′ sequences deposited in GenBank and BOLD, nearly a third (27%) are obviously erroneous—most likely due to species-level identification difficulties. This is an alarming situation, as incorrectly identified barcodes inevitably generate downstream problems.

## Data Availability

The molecular data presented in this study can be found in an online repository (National Genomics Data Center (NGDC, China). Open access depositories of the collection material (physical specimens and their genitalia mounts, including of all described new species) are listed in the article.
